# CircRNA-ceRNA Network Revealing the Potential Regulatory Roles of CircRNA in Alzheimer’s Disease Involved the cGMP-PKG Signal Pathway

**DOI:** 10.3389/fnmol.2021.665788

**Published:** 2021-05-21

**Authors:** Yuan Zhang, Lili Qian, Yingying Liu, Ying Liu, Wanpeng Yu, Yanfang Zhao

**Affiliations:** ^1^Institute for Translational Medicine, The Affiliated Hospital of Qingdao University, Qingdao University, Qingdao, China; ^2^Institute of Translational Medicine, The Affiliated Hospital of Hangzhou Normal University, Hangzhou, China; ^3^School of Basic Medical Sciences, Qingdao University, Qingdao, China; ^4^Institute of Biomedical Research, School for Life Sciences, Shandong University of Technology, Zibo, China

**Keywords:** Alzheimer’s disease, circRNA, hippocampus, expression profiles, ceRNA

## Abstract

**Background:** Alzheimer’s disease (AD) is a chronic progressive neurodegenerative disease. The characteristic pathologies include extracellular senile plaques formed by β-amyloid protein deposition, neurofibrillary tangles formed by hyperphosphorylation of tau protein, and neuronal loss with glial cell hyperplasia. Circular RNAs (circRNAs) are rich in miRNA-binding sites (miRNA response elements, MREs), which serve as miRNA sponges or competitive endogenous RNAs (ceRNAs). Although several research groups have identified dysregulated circRNAs in the cerebral cortex of SAMP8 mice or APP/PS1 mice using deep RNA-seq analysis, we need to further explore circRNA expression patterns, targets, functions and the signaling pathways involved in the pathogenesis of AD and in particular the hippocampal circRNA expression profiles in AD.

**Methods:** We used deep RNA sequencing to investigate circRNA-ceRNA network patterns in the hippocampus of APP/PS1 mice.

**Results:** In our study, 70 dysregulated circRNAs, 39 dysregulated miRNAs and 121 dysregulated mRNAs were identified between the APP/PS1 group and the wild-type group at 8 months in the hippocampus of the mice. Through correlation analysis, we identified 11 dysregulated circRNAs, 7 dysregulated miRNAs and 8 dysregulated mRNAs forming 16 relationships in the circRNA-miRNA-mRNA regulatory network. Gene ontology (GO) analysis indicated that the dysregulated circRNAs were most enriched in biological metabolic processes. Kyoto Encyclopedia of Genes and Genomes (KEGG) analysis showed that the dysregulation of circRNAs was enriched in the cGMP-PKG signaling pathway, cAMP signaling pathway, Hippo signaling pathway, platelet activation, long-term potentiation and axon guidance. In addition, our findings preliminarily verified that the novel_circ_0003012/mmu-miR-298-3p/Smoc2 signaling axis may regulate the pathophysiology of AD by affecting the cGMP-PKG signaling pathway.

**Conclusions:** These newly identified circRNAs in networks and signaling pathways reveal potential diagnostic or therapeutic targets for AD.

## Introduction

Alzheimer’s disease (AD) is a chronic progressive neurodegenerative disease and is the most common type of senile dementia (Tiwari et al., 2019). The main characteristics are memory impairment, cognitive decline, personality change and language impairment, which seriously affect people’s daily lives. However, the pathogenesis of AD has not been fully elucidated. The characteristic pathologies include extracellular senile plaques formed by β-amyloid protein deposition, neurofibrillary tangles formed by hyperphosphorylation of the tau protein, and neuronal loss with glial cell hyperplasia (Wang et al., 2014; Gouras et al., 2015; Wilkins and Swerdlow, 2017).

Circular RNA (circRNA) is a non-coding RNA with a unique covalent closed loop structure. CircRNAs are rich in miRNA-binding sites (miRNA response elements, MREs), which serve as miRNA sponges or competitive endogenous RNAs (ceRNAs) (Lei et al., 2018; Kristensen et al., 2019). Currently, several studies have shown that circRNAs play an important role in the regulation of neurodegenerative diseases via their interaction with disease-associated miRNAs (D’Ambra et al., 2019).

In previous studies, several research groups have identified dysregulated circRNAs in the cerebral cortex of SAMP8 mice or APP/PS1 mice using deep RNA-seq analysis (Zhang et al., 2017; Ma et al., 2019). Other groups have also identified dysregulated circRNAs in the hippocampal tissues of an AD mouse model by circRNA microarray (Huang et al., 2018; Wang et al., 2018). Currently, it is believed that the nerve loss caused by the development of AD is mainly in the cortex and hippocampus. The hippocampus is very important for learning and memory. Changes in the function and structure of the hippocampus are critical for learning and memory, such as long-term potentiation (LTP) and synaptic remodeling (Matsuzaki et al., 2004; Mu and Gage, 2011). Several key molecules influence the generation of new hippocampal neurons in AD, and significant changes in neurogenesis occur earlier than the onset of hallmark lesions or neuronal impairment (Lazarov and Marr, 2010).

Despite these findings, we need to further explore the expression patterns, targets, and functions of circRNAs and the signaling pathways involved in the pathogenesis of AD. Therefore, further research is of great importance. Here, we detected dysregulation of the circRNA-ceRNA profile in the hippocampus of APP/PS1 mice using deep RNA-seq analysis. We performed Gene Ontology (GO) and Kyoto Encyclopedia of Genes and Genomes (KEGG) analyses to predict the biological roles and potential signaling pathways of these differentially expressed circRNAs. Furthermore, we conducted circRNA-ceRNA network pattern analysis to further explore the potential roles of dysregulated circRNAs in AD pathogenesis. Taken together, our findings may promote a better understanding of the role of circRNAs in the neuropathogenesis of AD.

## Materials and Methods

### Animals

Eight-month-old APP/PS1 mice and their age-matched wild-type mice were purchased from Model Animal Research Institute of Myhalic (Wuhan, China). The mice were housed two per cage under the standard conditions (12 h light/dark cycle at 25°C and 50 ± 10% relative humidity). We randomly selected nine animals from each group, three animals for RNA-seq, and six animals for Real-time qPCR. Animals administered general anesthesia and then collected hippocampal tissue. Animal care and experimental procedures were implemented according to the document “Guidance Suggestions for Caring for Laboratory Animals” produced by the Ministry of Science and Technology of China in 2006.

### RNA Extraction and Qualification

Total RNA was extracted from each hippocampal tissue sample by RNAprep Pure Tissue Kit (TIANGEN BIOTECH, Beijing, China) in accordance with the manufacturer’s instructions.

Using 1% agarose gels to monitor RNA degradation and contamination. Then, using the NanoPhotometer^®^ spectrophotometer to check the RNA purity (IMPLEN, CA, United States) and using Qubit^®^ RNA Assay Kit in Qubit^®^ 2.0 Flurometer to measure the RNA concentration (Life Technologies, CA, United States). Finally, using the RNA Nano 6000 Assay Kit of the Bioanalyzer 2100 system to assess the RNA integrity (Agilent Technologies, CA, United States).

### RNA-Seq

Details of the mRNA-seq, miRNA-seq, and circRNA-seq methods are described in Supplementary Materials.

### Real-Time qPCR

To validate the RNA-Seq data, we randomly selected 3 of circRNA, miRNA and mRNA for qRT-PCR analysis, respectively. Total RNA was extracted from each hippocampal tissue sample, and then reverse-transcribed into cDNA using PrimeScript^TM^ RT reagent Kit with gDNA Eraser (Takara, Dalian, China) according to the manufacturer’s instruction.

Real-time quantitative PCR (RT-qPCR) was performed using the SYBR^®^ Premix Ex Taq^TM^ II (Tli RNase H Plus) Kit with a Bio-Rad CFX Manager 3.1 real-time PCR system (CFX96^TM^ Real-Time PCR, Bio-Rad, United States). The relative circRNA and mRNA expression levels were calculated using the 2^–ΔΔCt^ method and were normalized to GAPDH as an endogenous reference transcript. miRNA expression levels were normalized to that of U6. The specific primers for each gene are listed in Supplementary Table 1. Data shown represent the means of three experiments.

### GO Annotations and KEGG Pathway Analyses

Gene Ontology (GO) enrichment analysis of differentially expressed genes was conducted by clusterProfiler, an R package for functional classification and enrichment of gene clusters using hypergeometric distribution. KEGG is a database resource for understanding high-level functions and utilities of the biological system^1^. We used clusterProfiler R package to test the statistical enrichment of aberrantly expressed circRNAs in KEGG pathways. GO and KEGG terms with corrected *P*-value < 0.05 were considered significantly enrichment of aberrantly expressed circRNAs.

### Annotation for CircRNA-miRNA-mRNA Interaction

We have selected the differentially expressed circRNA, miRNA and mRNA that have been identified. CircRNA-miRNA interactions and miRNA-mRNA interactions were predicted with Arraystar’s home-made miRNA target prediction software based on TargetScan^2^ and miRanda^3^. The circRNA-miRNA-mRNA network covered two cases: upregulated circRNA-downregulated miRNA-upregulated mRNA, and downregulated circRNA-upregulated miRNA-upregulated mRNA. Then, we constructed circRNA-miRNA-mRNA network using the Cytoscape software V2.7.0 (San Diego, CA, United States).

### Statistical Analysis

Statistical analyses were performed using SPSS v16.0 software (SPSS, Inc., Chicago, IL, United States). All data were expressed as the mean ± SEM. *p* < 0.05 was statistically significant.

## Results

### Overview of CircRNA-Seq

A total of 514,529,568 raw reads were generated, 255,871,400 for wild-type (WT) mice, and 258,658,168 for APP/PS1 mice. Removed poly(N)-containing, low-quality, and adaptor-containing reads from the raw data, then remained 506,341,272 clean reads including 251,602,810 for wild-type and 254,738,462 for APP/PS1 mice. The high-quality clean data were mapped to the mouse reference sequence by Hisat2^4^ and the unmapped reads were subsequently selected (Pertea et al., 2016). circRNAs were detected and identified using find_circ and CIRI, 5,683 circRNAs were detected (Memczak et al., 2013; Gao et al., 2015). These circRNAs were used for subsequent analyses.

### Overview of miRNA-Seq

A total of 90,306,346 raw reads were generated, 42,973,163 for WT mice, and 47,333,183 for APP/PS1 mice. After removal of low-quality and adaptor sequences, 41,501,993 clean reads for WT group and 46,256,666 clean reads for APP/PS1 group were remained. The reads we selected are mostly based on the length of 21–22 nt in both groups. These reads were annotated and classified based on previous studies (Ma et al., 2019). Finally, 1,351 matured miRNAs (1,275 known and 76 novel) were detected. These miRNAs were used for the subsequent analysis.

### Overview of mRNA-Seq

A total of 267,484,154 raw reads were generated: 135,704,866 for APP/PS1 mice, and 131,779,288 for wild-type mice. After discarding the reads with adapters, poly-N > 10%, and discarding the low-quality reads. 211,535,266 UMI reads were obtained: 107,820,630 for APP/PS1 mice, and 103,714,636 for wild-type mice. The clean reads were mapped to the mouse reference genome, and the Dedup2MappedUMI rates were approximately 80.50% for APP/PS1 mice and 85.08% for wild-type mice. The cufflink results indicated that 57,077 protein-coding transcripts were identified. These mRNAs were used for the subsequent analysis.

### Differential Expression Analysis Between APP/PS1 and Wild-Type Mice

**FIGURE 1:**
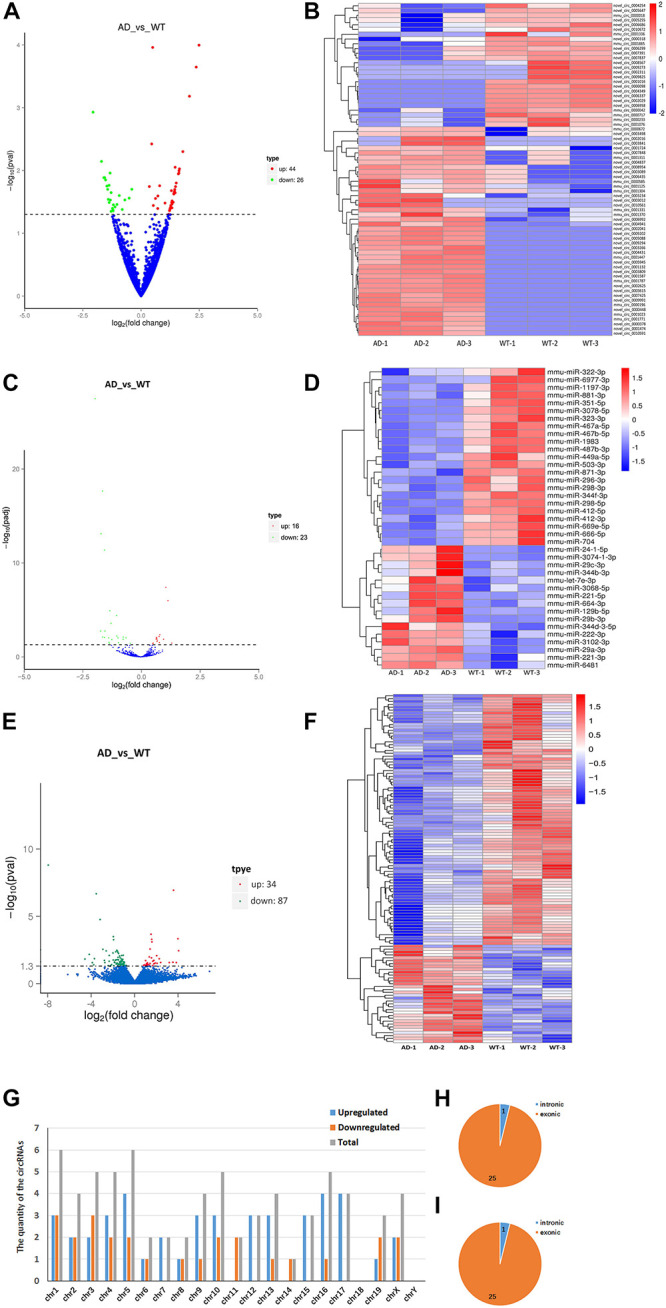
Expression profiles of distinct RNAs. **(A,B)** Expression profiles of circRNAs. **(A)** In the volcano plots, green, red, and blue points represent circRNAs that were downregulated, upregulated, and not significantly different in 8-month APP/PS1 mice relative to 8-month WT mice. **(B)** Cluster analysis of expression of circRNAs. Red and blue: increased and decreased expression, respectively. **(C,D)** Expression profiles of miRNAs. **(C)** In the volcano plots, green, red, and blue points represent miRNAs that were downregulated, upregulated, and not significantly different in 8-month APP/PS1 mice relative to 8-month WT mice. **(D)** Cluster analysis of expression of miRNAs. Red and blue: increased and decreased expression, respectively. **(E,F)** Expression profiles of mRNAs. **(E)** In the volcano plots, green, red, and blue points represent mRNAs that were downregulated, upregulated, and not significantly different in 8-month APP/PS1 mice relative to 8-month WT mice. **(F)** Cluster analysis of expression of mRNAs. Red and blue: increased and decreased expression, respectively. **(G–I)** Chromosomal distribution of differentially expressed circRNAs in hippocampal of APP/PS1 mice. **(G)** Chromosomal localization of differentially expressed circRNAs in hippocampal of APP/PS1 mice. **(H)** Gene localization of upregulated circRNAs in hippocampal of APP/PS1 mice. **(I)** Gene localization of downregulated circRNAs in hippocampal of APP/PS1 mice.

We identified 70 significantly aberrantly expressed circRNAs between APP/PS1 mice and wild-type (WT) mice at 8 months in the hippocampus (*p* < 0.05), of which 44 circRNAs were upregulated and 26 were downregulated (Table 1). We performed cluster analysis on the differential circRNA expression and generated a heatmap to visualize the results of the cluster analysis (Figures 1A,B).

**TABLE 1:** Differently expressed circRNAs between APP/PS1 and WT mice.

**Name**	***p*-value**	**log2Fold Change**	**circRNA type**	**Chr.**	**Source gene name**
**Up-regulated**					
mmu_circ_0001787	0.00010101	2.4893	Exonic	chr9	Glce
novel_circ_0001132	0.0002256	2.3685	Intronic	chr12	Unc79
novel_circ_0003089	0.0006568	2.079	Exonic	chr16	Mapk1
novel_circ_0005088	0.0050106	1.7954	Exonic	chr1	Phf3
mmu_circ_0000196	0.0094188	1.6488	Exonic	chr10	Ano4
mmu_circ_0001771	0.010104	1.631	Exonic	chr9	Cadm1
novel_circ_0007848	0.011021	1.6252	Intronic	chr5	Fam193a
novel_circ_0001474	0.011332	1.602	Exonic	chr12	Mipol1
novel_circ_0003841	0.015839	1.5111	Intronic	chr17	Nrxn1
novel_circ_0005945	0.01807	1.4807	Exonic	chr2	Rc3h2
novel_circ_0008954	0.022687	1.4658	Exonic	chr7	Gas2
novel_circ_0003498	0.008883	1.4639	Exonic	chr17	Telo2
novel_circ_0002016	0.020061	1.4473	Exonic	chr13	2210408I21Rik
novel_circ_0003615	0.021294	1.437	Exonic	chr17	St6gal2
novel_circ_0001587	0.02155	1.4335	Intronic	chr12	Sipa1l1
novel_circ_0000448	0.021844	1.4298	Exonic	chr10	Plxnc1
novel_circ_0009294	0.022252	1.4243	Exonic	chr8	Pdpr
novel_circ_0002041	0.022454	1.4216	Exonic	chr13	Wdr37
novel_circ_0007425	0.022994	1.4151	Exonic	chr4	Atg4c
novel_circ_0004431	0.023718	1.4056	Exonic	chr19	Slf2
novel_circ_0004837	0.032011	1.3795	Exonic	chr1	Dcaf6
novel_circ_0009991	0.030549	1.3331	Exonic	chr9	Cdon
novel_circ_0001724	0.034337	1.3294	Exonic	chr13	Ipo11
novel_circ_0006435	0.03936	1.3265	Exonic	chr3	Miga1
novel_circ_0010591	0.032498	1.3142	Exonic	chrX	Ctps2
mmu_circ_0000672	0.02096	1.3038	Exonic	chr16	Pi4ka
novel_circ_0010561	0.036087	1.2772	Exonic	chrX	Cnksr2
novel_circ_0009202	0.039016	1.259	Exonic	chr7	Picalm
mmu_circ_0001447	0.040307	1.2485	Exonic	chr6	Cped1
novel_circ_0003266	0.040406	1.2478	Exonic	chr16	Lsamp
mmu_circ_0001023	0.041613	1.2385	Exonic	chr2	Tank
novel_circ_0000378	0.04377	1.2224	Exonic	chr10	Slc41a2
novel_circ_0003012	0.043524	1.219	Exonic	chr15	Adcy6
novel_circ_0002625	0.049801	1.1821	Exonic	chr15	Npr3
novel_circ_0003809	0.049842	1.1819	Exonic	chr17	Srbd1
novel_circ_0004941	0.033054	1.0292	Exonic	chr1	Cdc42bpa
novel_circ_0003234	0.017509	0.79777	Exonic	chr16	Stxbp5l
mmu_circ_0001370	0.025479	0.72313	Exonic	chr5	Cds1
mmu_circ_0001304	0.040342	0.71604	Exonic	chr4	Rere
novel_circ_0006992	0.02793	0.61947	Exonic	chr4	Ptp4a2
mmu_circ_0000585	0.036122	0.52692	Exonic	chr15	Stk3
mmu_circ_0001125	0.00010971	0.49891	Exonic	chr3	Elf2
mmu_circ_0001331	0.0037768	0.45734	Exonic	chr5	Ppp1cb
mmu_circ_0001311	0.01803	0.35681	Exonic	chr5	Ankib1
**Down-regulated**					
mmu_circ_0000042	0.0011776	−2.0607	Exonic	chr1	Plcl1
novel_circ_0006337	0.0071747	−1.6991	Exonic	chr3	Tmem56
novel_circ_0007391	0.013014	−1.5984	Exonic	chr4	Focad
novel_circ_0007837	0.012984	−1.5635	Exonic	chr5	Nsd2
novel_circ_0002311	0.014154	−1.5245	Exonic	chr14	Vdac2
novel_circ_0004349	0.017713	−1.4676	Exonic	chr19	Btaf1
novel_circ_0000098	0.019977	−1.4339	Exonic	chr10	Usp15
novel_circ_0005647	0.028912	−1.4057	Exonic	chr2	Ralgapa2
mmu_circ_0000233	0.017241	−1.3738	Exonic	chr11	Tns3
novel_circ_0000318	0.032366	−1.3682	Exonic	chr10	Arid5b
novel_circ_0008567	0.010996	−1.3302	Intronic	chr6	Ctnna2
novel_circ_0009825	0.029543	−1.3182	Exonic	chr9	Qrich1
novel_circ_0006299	0.044778	−1.2889	Exonic	chr3	Vav3
mmu_circ_0001865	0.022528	−1.2856	Exonic	chrX	Tspan7
novel_circ_0001016	0.040106	−1.2277	Exonic	chr11	Ppm1d
novel_circ_0009273	0.042078	−1.2096	Exonic	chr8	Nfatc3
novel_circ_0002029	0.043894	−1.1991	Exonic	chr13	Adarb2
novel_circ_0006958	0.043894	−1.1991	Exonic	chr4	Zmym4
novel_circ_0005255	0.03522	−1.1883	Exonic	chr1	Kansl1l
mmu_circ_0000018	0.03809	−1.1185	Exonic	chr1	Rims1
novel_circ_0004254	0.03478	−1.035	Exonic	chr19	Trpm3
novel_circ_0010672	0.025091	−0.93642	Exonic	chrX	–
novel_circ_0006686	0.041843	−0.8083	Exonic	chr3	Wdr49
mmu_circ_0000717	0.029471	−0.69561	Exonic	chr16	Ttc3
mmu_circ_0001076	0.027567	−0.51113	Exonic	chr2	Tasp1
mmu_circ_0001336	0.019943	−0.39615	Exonic	chr5	Nsd2

Next, we identified 39 significantly aberrantly expressed miRNAs between APP/PS1 mice and WT mice (*p* < 0.05), of which 16 miRNAs were upregulated and 23 were downregulated (Table 2). Cluster analysis and heatmapping were performed to show the results of the differential miRNA expression (Figures 1C,D).

**TABLE 2:** Differently expressed miRNAs between APP/PS1 and WT mice.

**Name**	**AD_ readcount**	**Ctrl_ readcount**	**log2FoldChange**	***p*-value**
**Up-regulated**				
mmu-miR-6481	18.68982	4.322652	1.3238	0.001602
mmu-miR-29b-3p	495.2749	211.0125	1.1645	8.24E-09
mmu-miR-29a-3p	118111.2	54394.02	1.0745	2.69E-10
mmu-miR-344d-3-5p	319.5235	148.8742	0.97566	0.000481
mmu-miR-3074-1-3p	185.8845	102.5404	0.8147	7.04E-05
mmu-miR-29c-3p	139.5045	76.32593	0.7979	0.001251
mmu-miR-24-1-5p	188.332	105.8336	0.78842	0.000132
mmu-miR-129b-5p	2732.962	1536.956	0.78262	0.000131
mmu-miR-3102-3p	962.5197	569.701	0.71115	0.000643
mmu-miR-222-3p	18468.63	11009.11	0.69991	0.000922
mmu-miR-664-3p	1197.966	725.9918	0.68802	0.000265
mmu-miR-221-5p	2240.683	1361.592	0.68249	0.000392
mmu-miR-3068-5p	340.8997	209.7466	0.66796	0.000222
mmu-miR-221-3p	16177.18	10037.58	0.65584	0.00042
mmu-let-7e-3p	405.1511	275.5497	0.53313	0.001513
mmu-miR-344b-3p	1073.644	738.0082	0.52116	0.00206
**Down-regulated**				
mmu-miR-298-5p	333.5885	1352.94	−1.9447	4.46E-31
mmu-miR-1197-3p	4.053018	23.29665	−1.7052	2.61E-05
mmu-miR-1983	392.6913	1361.133	−1.6874	3.13E-16
mmu-miR-412-5p	473.496	1532.112	−1.6259	5.95E-21
mmu-miR-298-3p	2.126654	17.24759	−1.6224	0.000178
mmu-miR-344f-3p	155.3415	482.7177	−1.5446	2.33E-14
mmu-miR-881-3p	4.187014	22.05614	−1.5438	0.000216
mmu-miR-449a-5p	12.80265	50.6019	−1.5218	2.61E-05
mmu-miR-871-3p	9.149676	31.4921	−1.3645	0.000292
mmu-miR-669e-5p	3.739892	16.45383	−1.35	0.001142
mmu-miR-6977-3p	4.825787	22.62498	−1.3302	0.001815
mmu-miR-666-5p	918.8775	2496.094	−1.3144	1.17E-07
mmu-miR-296-3p	126.8954	339.053	−1.2638	3.31E-06
mmu-miR-704	11.4574	33.68301	−1.2305	0.000545
mmu-miR-323-3p	3855.472	8182.974	−1.0241	4.28E-07
mmu-miR-503-3p	34.76984	76.19848	−1.0154	0.000105
mmu-miR-412-3p	108.1681	228.1667	−0.96854	0.000283
mmu-miR-322-3p	262.6622	558.4182	−0.94468	0.001368
mmu-miR-3078-5p	73.46593	127.4599	−0.75059	0.000458
mmu-miR-351-5p	221.2018	378.9669	−0.73994	0.000168
mmu-miR-487b-3p	1994.151	3127.145	−0.62393	0.000249
mmu-miR-467a-5p	901.2772	1233.437	−0.44142	0.001861
mmu-miR-467b-5p	901.2772	1233.437	−0.44142	0.001861

Finally, we estimated the expression levels of the mRNA transcripts. A total of 121 mRNAs were significantly aberrantly expressed between the APP/PS1 mice and the WT mice (*p* < 0.05), with 34 upregulated mRNAs and 87 downregulated mRNAs (Table 3). Cluster analysis and heatmapping were performed to show the results of the differential mRNA expression (Figures 1E,F).

**TABLE 3:** Differently expressed mRNAs between APP/PS1 and WT mice.

**Gene_id**	**Readcount_AD**	**Readcount_Ctrl**	**Log2FoldChange**	***p*-value**	**Gene name**
**Up-regulated**					
ENSMUSG00000081229	59.69713	0	Inf	4.76E-15	Lamr1-ps1
ENSMUSG00000083481	9.956849	0.597699	4.0582	0.0035986	Rps8-ps2
ENSMUSG00000068129	14.61925	0.919661	3.9906	0.00046637	Cst7
ENSMUSG00000097754	8.600735	0.599123	3.8435	0.027421	Ptgs2os2
ENSMUSG00000104674	55.02875	4.59332	3.5826	1.16E-07	Gm42756
ENSMUSG00000098758	10.60356	0.918237	3.5295	0.026071	7630403G23Rik
ENSMUSG00000081738	134.6703	14.72345	3.1932	0.027496	Hmgb1-ps2
Novel01171	13.22348	2.500661	2.4027	0.027206	−//−
Novel01252	27.8467	5.773438	2.27	0.014262	–
ENSMUSG00000006179	12.82333	3.055696	2.0692	0.030813	Prss16
Novel00437	23.62582	5.769878	2.0338	0.0083936	–
Novel00379	51.25592	13.7437	1.8989	0.029936	–
Novel01429	21.80691	6.345889	1.7809	0.033866	–
ENSMUSG00000096256	27.55918	8.075859	1.7708	0.019005	Gm21093
Novel01018	36.84361	11.54332	1.6744	0.021943	−//−
ENSMUSG00000109588	33.27743	10.43325	1.6734	0.045052	Lnp1
ENSMUSG00000041828	160.808	53.22581	1.5951	0.00079777	Abca8a
ENSMUSG00000024810	659.4621	221.4448	1.5743	0.00051526	Il33
ENSMUSG00000110027	65.12381	22.14243	1.5564	0.012239	C030029H02Rik
ENSMUSG00000079037	28336.45	9928.219	1.5131	0.00021683	Prnp
ENSMUSG00000084159	37.20414	13.89054	1.4214	0.031861	Gm12696
ENSMUSG00000064201	249.4366	93.2043	1.4202	0.0027087	Krt2
ENSMUSG00000027173	50.06709	19.49118	1.361	0.042989	Depdc7
ENSMUSG00000022892	90474.64	35410.63	1.3533	0.010394	App
Novel00624	52.06992	20.71021	1.3301	0.04299	−//−
ENSMUSG00000054986	51.03505	21.26117	1.2633	0.039106	Sec14l3
ENSMUSG00000105891	38.45599	16.56134	1.2154	0.035959	A230001M10Rik
ENSMUSG00000063902	297.1976	131.3646	1.1778	0.028533	Gm7964
ENSMUSG00000095690	44.8563	20.49973	1.1297	0.0425	Rab11b-ps2
ENSMUSG00000069516	93.97817	44.04001	1.0935	0.033869	Lyz2
ENSMUSG00000021732	167.113	81.39735	1.0378	0.042625	Fgf10
ENSMUSG00000062825	13041.13	6475.64	1.01	0.01957	Actg1
ENSMUSG00000020251	240.9446	127.1682	0.92196	0.041038	Glt8d2
ENSMUSG00000067288	392.7952	223.849	0.81125	0.044513	Rps28
**Down-regulated**					
ENSMUSG00000024903	0	7.882801	−Inf	0.0030367	Lao1
ENSMUSG00000053441	0	5.745342	−Inf	0.024105	Adamts19
Novel00685	0.565765	139.4277	−7.9451	1.57E-09	−//−
ENSMUSG00000047773	0.565765	13.41798	−4.5678	0.015067	Ankfn1
ENSMUSG00000118133	0.565765	10.16852	−4.1678	0.006811	AC102268.2
ENSMUSG00000070473	0.516452	7.667856	−3.8921	0.028073	Cldn3
Novel01330	0.516452	7.007228	−3.7621	0.04024	−
ENSMUSG00000025383	0.774678	10.01955	−3.6931	0.014138	Il23a
ENSMUSG00000035551	7.033407	81.43574	−3.5334	2.10E-07	Igfbpl1
ENSMUSG00000004328	5.915878	53.47947	−3.1763	1.73E-05	Hif3a
ENSMUSG00000115529	7.914695	60.06239	−2.9239	0.0028139	9630013A20Rik
ENSMUSG00000074217	1.955521	13.45871	−2.7829	0.019075	2210011C24Rik
ENSMUSG00000049598	1.746609	11.60482	−2.7321	0.027804	Vsig8
ENSMUSG00000047109	3.934697	24.22868	−2.6224	0.0037399	Cldn14
ENSMUSG00000057606	1.929862	10.4023	−2.4303	0.045634	Colq
ENSMUSG00000027654	9.478051	48.00599	−2.3406	0.021837	Fam83d
ENSMUSG00000051980	2.357676	11.84359	−2.3287	0.008081	Casr
ENSMUSG00000026435	3.037738	14.20985	−2.2258	0.047127	Slc45a3
ENSMUSG00000104494	12.53744	54.5147	−2.1204	0.039037	Gm37111
ENSMUSG00000028661	12.59477	50.02412	−1.9898	0.0053336	Epha8
ENSMUSG00000017697	4.731023	18.54485	−1.9708	0.017823	Ada
ENSMUSG00000029675	51.15106	198.07	−1.9532	0.0003248	Eln
ENSMUSG00000026043	20.30419	77.41927	−1.9309	0.00054551	Col3a1
ENSMUSG00000023153	3.958351	14.95509	−1.9177	0.046526	Tmem52
ENSMUSG00000066407	6.51495	23.67792	−1.8617	0.025408	Gm10263
ENSMUSG00000025270	16.99661	59.72416	−1.8131	0.027639	Alas2
ENSMUSG00000040298	9.491716	32.54046	−1.7775	0.01854	Btbd16
ENSMUSG00000029005	10.42031	34.6719	−1.7344	0.0043488	Draxin
ENSMUSG00000022090	88.96601	287.1362	−1.6904	0.035372	Pdlim2
ENSMUSG00000032278	13.27847	42.62864	−1.6827	0.017123	Paqr5
ENSMUSG00000069917	17.62335	55.95631	−1.6668	0.017287	Hba-a2
ENSMUSG00000027570	195.4526	609.5565	−1.6409	0.0033892	Col9a3
ENSMUSG00000036913	13.19585	40.75662	−1.6269	0.029634	Trim67
ENSMUSG00000026347	85.99188	261.2666	−1.6033	0.030127	Tmem163
ENSMUSG00000015202	40.48648	122.0282	−1.5917	0.003746	Cnksr3
ENSMUSG00000039328	41.31611	121.2591	−1.5533	0.041664	Rnf122
ENSMUSG00000026879	856.6371	2497.441	−1.5437	0.0046071	Gsn
ENSMUSG00000037295	24.41851	70.57507	−1.5312	0.011442	Ldlrap1
ENSMUSG00000021835	19.88039	57.41643	−1.5301	0.016712	Bmp4
ENSMUSG00000052688	16.68903	47.42609	−1.5068	0.017068	Rab7b
ENSMUSG00000079017	8.727027	24.66459	−1.4989	0.034856	Ifi27l2a
ENSMUSG00000027750	12.84297	36.12737	−1.4921	0.01735	Postn
ENSMUSG00000027669	95.09477	264.4664	−1.4756	0.0057058	Gnb4
ENSMUSG00000043903	14.93845	40.98431	−1.456	0.037328	Zfp469
ENSMUSG00000027858	972.4845	2549.279	−1.3903	0.035674	Tspan2
ENSMUSG00000031285	64.95229	169.2304	−1.3815	0.0088063	Dcx
ENSMUSG00000046718	47.23032	122.6923	−1.3773	0.024186	Bst2
ENSMUSG00000083061	26.84117	69.17754	−1.3659	0.027105	Gm12191
ENSMUSG00000076439	604.8117	1557.412	−1.3646	0.038822	Mog
ENSMUSG00000040373	66.88182	169.6338	−1.3427	0.0119	Cacng5
ENSMUSG00000052305	55.29137	137.6412	−1.3158	0.026801	Hbb-bs
ENSMUSG00000049928	22.40201	54.60002	−1.2853	0.04063	Glp2r
ENSMUSG00000095123	23.96537	57.53148	−1.2634	0.043398	Gm21781
ENSMUSG00000038550	56.48149	135.2756	−1.2601	0.020549	Ciart
ENSMUSG00000055415	21.08322	50.34414	−1.2557	0.034212	Atp10b
ENSMUSG00000049721	60.25462	142.5146	−1.242	0.033117	Gal3st1
ENSMUSG00000029661	86.15144	201.1932	−1.2236	0.014912	Col1a2
ENSMUSG00000028655	117.6374	270.6833	−1.2023	0.024522	Mfsd2a
ENSMUSG00000056999	291.2056	665.5641	−1.1925	0.0081406	Ide
ENSMUSG00000061436	173.1805	391.0994	−1.1753	0.035685	Hipk2
ENSMUSG00000029622	149.4121	334.9681	−1.1647	0.019696	Arpc1b
ENSMUSG00000029570	90.59498	200.9065	−1.149	0.035382	Lfng
ENSMUSG00000105987	33.82957	73.99915	−1.1292	0.034879	AI506816
ENSMUSG00000055485	169.7093	370.7928	−1.1275	0.018528	Soga1
ENSMUSG00000054404	72.10059	156.4623	−1.1177	0.038152	Slfn5
ENSMUSG00000022197	300.6014	640.066	−1.0904	0.015608	Pdzd2
ENSMUSG00000038375	876.3089	1862.118	−1.0874	0.036051	Trp53inp2
ENSMUSG00000021903	23.37557	49.0625	−1.0696	0.048392	Galnt15
ENSMUSG00000102234	57.9682	121.1882	−1.0639	0.048061	Gm37885
ENSMUSG00000038173	110.4855	230.3017	−1.0597	0.047454	Enpp6
ENSMUSG00000038059	80.18825	167.0305	−1.0586	0.03448	Smim3
ENSMUSG00000052229	619.3117	1275.477	−1.0423	0.0078224	Gpr17
ENSMUSG00000033685	218.4847	448.8172	−1.0386	0.04342	Ucp2
ENSMUSG00000020661	433.9094	890.5794	−1.0374	0.023838	Dnmt3a
ENSMUSG00000028962	262.8775	538.6218	−1.0349	0.035006	Slc4a2
ENSMUSG00000022096	152.228	311.8075	−1.0344	0.047777	Hr
ENSMUSG00000030168	472.0721	966.8106	−1.0342	0.029204	Adipor2
ENSMUSG00000033209	110.2507	219.8822	−0.99594	0.04941	Ttc28
ENSMUSG00000049791	58.0428	115.4981	−0.99268	0.047269	Fzd4
ENSMUSG00000035104	92.2629	182.6223	−0.98504	0.047666	Eva1a
ENSMUSG00000029086	96.90165	190.6386	−0.97625	0.049665	Prom1
ENSMUSG00000040268	743.4839	1454.211	−0.96786	0.031958	Plekha1
ENSMUSG00000021614	134.8811	257.3204	−0.93188	0.025777	Vcan
ENSMUSG00000030711	51.07835	97.36197	−0.93065	0.043666	Sult1a1
ENSMUSG00000036036	31.18596	57.70265	−0.88774	0.045222	Zfp57
ENSMUSG00000006403	348.0064	602.54	−0.79194	0.048289	Adamts4
ENSMUSG00000040565	264.231	452.6413	−0.77657	0.044065	Btaf1

The data showed that the significantly aberrantly expressed circRNAs were scattered across different chromosomes: the 44 upregulated circRNAs were located on 17 chromosomes, and the 26 downregulated circRNAs were located on 15 chromosomes. The top three chromosomes for the upregulated circRNAs were chromosome (chr.) 5 (4/44), chr. 16 (4/44), and chr. 17 (4/44), while the top two chromosomes for the downregulated circRNAs were chr. 1 (3/26) and chr. 3 (3/26). As for localization of the dysregulated circRNAs, there were 40 exonic and 4 intronic in the upregulated circRNAs and 25 exonic and 1 intronic in the downregulated circRNAs (Figures 1G–I and Table 1).

### qPCR Confirmation

**FIGURE 2:**
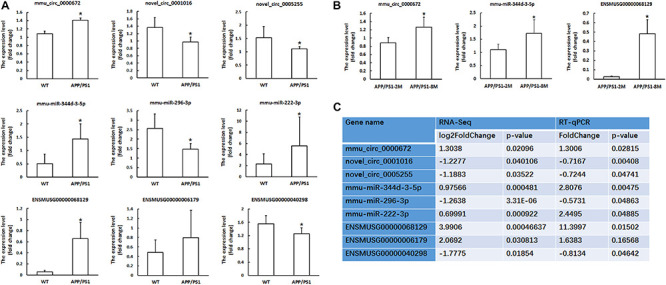
Validation of circRNA, miRNA and mRNAs expression by qPCR. **(A)** The expression levels of candidate circRNAs, miRNAs and mRNAs for validation by qPCR in hippocampal tissues of 8-month-old APP/PS1 mice and WT mice. **(B)** The expression levels of candidate circRNAs, miRNAs and mRNAs for validation by qPCR in hippocampal tissues of 8-month-old APP/PS1 mice and 2-month-old APP/PS1 mice. CircRNA and mRNA expression was quantified relative to Gapdh expression level by using the comparative cycle threshold (ΔCT) method. MiRNA expression was quantified relative to U6 expression level by using the comparative cycle threshold (ΔCT) method. Data are presented as means ± SD (*n* = 6; *p < 0.05). **(C)** The comparison information of the RNA-Seq and qRT-PCR data of 8-month-old APP/PS1 mice vs. WT mice.

We used RT-qPCR to confirm the differentially expressed RNAs in our RNA-seq experiments. We randomly selected three circRNAs, three miRNAs and three mRNAs to perform RT-qPCR. As shown in Figure 2A, all selected transcripts were detected in the hippocampus of the APP/PS1 mice and WT mice and nearly exhibited significant differential expression. In summary, near consistency was observed between the qPCR results and the RNA-seq data.

Furthermore, we confirmed the differential expression of circRNAs, miRNAs and mRNAs in 8-month-old APP/PS1 mice relative to 2-month-old APP/PS1 mice. The results showed that mmu_circ_0000672, mmu-miR-344d-3-5p and ENSMUSG00000068129 (*Cst7*) were significantly different between the hippocampal tissues of 8-month-old APP/PS1 mice and 2-month-old APP/PS1 mice (*P* < 0.05) (Figure 2B). This result indicated that these genes changed significantly with the age of the APP/PS1 mice.

### GO and KEGG Analyses

**FIGURE 3:**
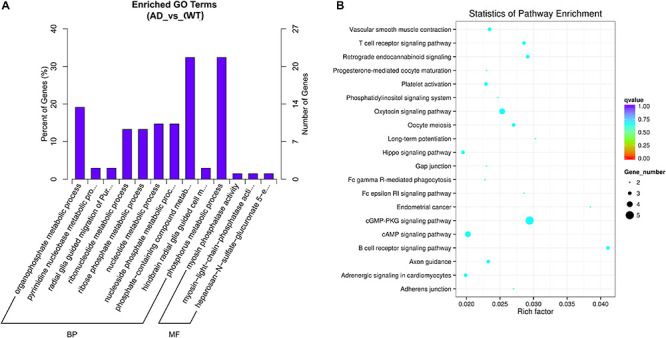
**(A)** Gene Ontology (GO) Enrichment Annotations of pathological progression of AD: Biological Process (BP) and Molecular Function (MF). **(B)** Significantly Enriched Kyoto Encyclopedia of Genes and Genomes (KEGG). The aberrantly expressed circRNAs in distinct aspects of AD pathology.

Gene Ontology (GO) analyses were performed on the circRNAs, and the top highly significantly enriched GO terms of the dysregulated circRNAs on biological process (BP) and molecular function (MF) are shown in Figure 3A. The 5 top terms were phosphorus metabolic process (GO: 0006793), phosphate-containing compound metabolic process (GO: 0006796),

organophosphate metabolic process (GO: 0019637), nucleotide metabolic process (GO: 0009117) and nucleoside phosphate metabolic process (GO: 0006753). Several metabolic pathway-related terms were also observed, such as pyrimidine nucleobase metabolic process (GO: 0006206), ribonucleotide metabolic process (GO: 0009259) and ribose phosphate metabolic process (GO: 0019693). In summary, the pathological progression of AD may be associated with several metabolic pathways regulated by circRNAs.

In addition, we also performed GO analysis of miRNAs and mRNAs. Through GO analysis of miRNAs, we found that the 20 top terms enriched in BP, cellular component (CC) and MF were almost all associated with cellular metabolic process, intracellular organelle/part and binding functions (Supplementary Figure 1A): cellular metabolic process (GO: 0044237), metabolic process (GO: 0008152), cellular macromolecule metabolic process (GO: 0044260), intracellular part (GO: 0044424), intracellular organelle (GO: 0043229), membrane-bounded organelle (GO: 0043227), intracellular membrane-bounded organelle (GO:0043231), protein binding (GO:0005515), and binding (GO: 0005488). Moreover, GO analysis indicated that the most enriched mRNAs correlated with single-organism developmental process (GO: 0044767), developmental process (GO: 0032502), multicellular organismal development (GO: 0007275), anatomical structure development (GO: 0048856), system development (GO: 0048731), gliogenesis (GO:0042063), extracellular region (GO: 0005576), extracellular region part (GO: 0044421), extracellular space (GO: 0005615) and structural molecule activity (GO: 0005198) (Supplementary Figure 2A). This result indicated that the dysregulated mRNAs were mostly enriched in the cellular/organism development process or cell differentiation.

Kyoto Encyclopedia of Genes and Genomes pathway analysis was performed to determine the signaling pathways related to the dysregulated circRNAs. By using the Q-value scale from 0 to 1, the top 20 significantly enriched pathways were identified, as shown in Figure 3B. Specifically, the cGMP-PKG signaling pathway, cAMP signaling pathway, axon guidance, platelet activation, LTP, Hippo signaling pathway and phosphatidylinositol signaling system were demonstrated to be closely related to the onset and development of AD.

Kyoto Encyclopedia of Genes and Genomes pathways were associated with dysregulated miRNAs involved in the MAPK signaling pathway, Ras signaling pathway, endocytosis, focal adhesion, axon guidance, neurotrophin signaling pathway and glycerophospholipid metabolism (Supplementary Figure 1B). KEGG pathway analysis of the dysregulated mRNAs identified enrichment in metabolic pathways, protein digestion and absorption, ribosomes, regulation of actin cytoskeleton, PI3K-Akt signaling pathway, platelet activation, spliceosome, tight junction, and the Hippo signaling pathway (Supplementary Figure 2B).

### Construction of the CircRNA-ceRNA Regulatory Networks

CircRNAs have MREs, which can be used as miRNA sponges to competitively bind miRNAs, thereby inhibiting miRNA targets to mRNA and indirectly regulating mRNA expression. Based on ceRNA theory, to search for circRNA-miRNA-gene pairs with the same MREs, circRNA-miRNA-gene pairs were constructed with the circRNA as a decoy, the miRNA as the core, and the mRNA as the target to construct a ceRNA regulatory network. The circRNA-ceRNA network pattern can show the regulation of the circRNA on the related mRNA-encoding genes.

**FIGURE 4:**
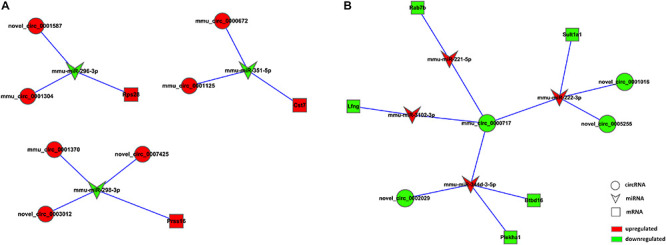
CircRNA-ceRNA network analysis in hippocampal tissue of APP/PS1 Mice. The ceRNA networks were based on circRNA-miRNA and miRNA-mRNA interactions. Circle nodes represent circRNAs, triangle nodes represent miRNAs, and rectangle nodes represent mRNAs. Red represent upregulated, green represent downregulated. **(A)** circRNA (down in APP/PS1 mice)-miRNA (up in APP/PS1 mice)-mRNA (down in APP/PS1 mice). **(B)** circRNA (up in APP/PS1 mice)-miRNA (down in APP/PS1 mice)-mRNA (up in APP/PS1 mice).

Based on the RNA-seq data, we selected 11 dysregulated circRNAs, 7 dysregulated miRNAs and 8 dysregulated mRNAs, and there were 16 relationships contained in the constructed circRNA-miRNA-mRNA regulatory network (Figure 4 and Table 4). The ceRNA network covered two cases: one was circRNA (7 circRNAs upregulated in APP/PS1 mice)-miRNA (3 miRNAs downregulated in APP/PS1 mice)-mRNA (3 mRNAs upregulated in APP/PS1 mice), and the other was circRNA (4 circRNAs downregulated)-miRNA (4 miRNAs upregulated)-mRNA (5 mRNAs upregulated). These circRNA-miRNA-mRNA interactions may supply a novel perspective for the pathogenesis of AD. We observed that one circRNA could interact with different miRNAs and that one miRNA could be regulated by multiple circRNAs; for example, mmu_circ_0000717 could interact with mmu-miR-222-3p, mmu-miR-221-5p, mmu-miR-3102-3p and mmu-miR-344d-3-5p, and mmu-miR-298-3 could co-associate with mmu_circ_0001370, novel_circ_0007425, and novel_circ_0003012.

**TABLE 4:** CircRNA-ceRNA networks in AD.

**CircRNA name**	**MiRNA name**	**Gene_id**	**Gene name**
mmu_circ_0001304	mmu-miR-296-3p	ENSMUSG00000067288	Rps28
novel_circ_0001587	mmu-miR-296-3p	ENSMUSG00000067288	Rps28
mmu_circ_0000672	mmu-miR-351-5p	ENSMUSG00000068129	Cst7
mmu_circ_0001125	mmu-miR-351-5p	ENSMUSG00000068129	Cst7
novel_circ_0003012	mmu-miR-298-3p	ENSMUSG00000006179	Prss16
novel_circ_0007425	mmu-miR-298-3p	ENSMUSG00000006179	Prss16
mmu_circ_0001370	mmu-miR-298-3p	ENSMUSG00000006179	Prss16
mmu_circ_0000717	mmu-miR-3102-3p	ENSMUSG00000029570	Lfng
mmu_circ_0000717	mmu-miR-221-5p	ENSMUSG00000052688	Rab7b
mmu_circ_0000717	mmu-miR-344d-3-5p	ENSMUSG00000040268	Plekha1
mmu_circ_0000717	mmu-miR-344d-3-5p	ENSMUSG00000040298	Btbd16
novel_circ_0002029	mmu-miR-344d-3-5p	ENSMUSG00000040268	Plekha1
novel_circ_0002029	mmu-miR-344d-3-5p	ENSMUSG00000040298	Btbd16
mmu_circ_0000717	mmu-miR-222-3p	ENSMUSG00000030711	Sult1a1
novel_circ_0005255	mmu-miR-222-3p	ENSMUSG00000030711	Sult1a1
novel_circ_0001016	mmu-miR-222-3p	ENSMUSG00000030711	Sult1a1

### Verification of the Potential Regulatory Mechanism of circRNAs in the Key Signaling Pathways

Through KEGG analysis, we obtained key regulatory signaling pathways, including the cGMP-PKG signaling pathway, cAMP signaling pathway, and Hippo signaling pathway. These pathways have been reported to participate in key regulatory roles in neurodegenerative diseases.

We further explored the regulatory effects of the differential expression of circRNAs on the cGMP-PKG signaling pathway, cAMP signaling pathway, and Hippo signaling pathway. We searched for the differentially expressed circRNAs enriched in the 3 signaling pathways and obtained five circRNAs that might be involved in the cGMP-PKG signaling pathway: novel_circ_0002311, novel_circ_0009273, novel_circ_0003012, novel_circ_0003089, and novel_circ_0001331. Four circRNAs might be involved in the cAMP signaling pathway: novel_circ_0006299, novel_circ_0003012, novel_circ_0003089, and novel_circ_0001331. Two circRNAs might be involved in the Hippo signaling pathway: novel_circ_0008567 and mmu_circ_0000585.

We predicted differentially expressed miRNAs that interact with those circRNAs and found that novel_circ_0009273, novel_circ_0003012, novel_circ_0006299, novel_circ_0008567, and mmu_circ_0000585 could target mmu-miR-3074-1-3p, mmu-miR-298-3p, mmu-miR-296-3p, mmu-miR-298-5p, mmu-miR-3074-1-3p, and mmu-miR-669e-5p, respectively.

**FIGURE 5:**
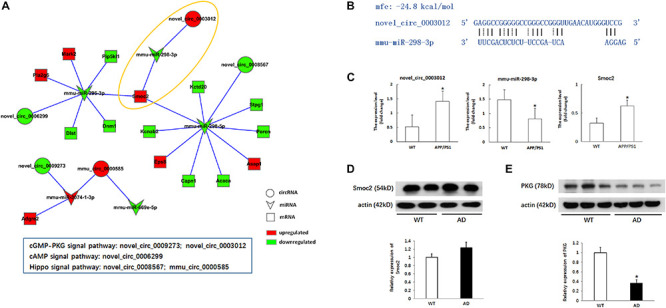
Verification of the potential regulation mechanism of circRNAs in the key signaling pathways. **(A)** CircRNA-ceRNA network of circRNAs in the key signaling pathways. Circle nodes represent circRNAs, triangle nodes represent miRNAs, and rectangle nodes represent mRNAs. Red represent upregulated, green represent downregulated. **(B)** The interaction of novel_circ_0003012/mmu-miR-298-3p. **(C)** The expression levels of novel_circ_0003012, mmu-miR-298-3p and SMOC2 by qPCR in hippocampal tissues of 8-month-old APP/PS1 mice and WT mice. CircRNA and mRNA expression was quantified relative to Gapdh expression level by using the comparative cycle threshold (ΔCT) method. MiRNA expression was quantified relative to U6 expression level by using the comparative cycle threshold (ΔCT) method. Data are presented as means ± SD (*n* = 6; *p < 0.05). **(D)** The expression of Smoc2 by Western blot in hippocampal tissues of 8-month-old APP/PS1 mice and WT mice. Data are presented as means ± SD (*n* = 2). **(E)** The expression of PKG by Western blot in hippocampal tissues of 8-month-old APP/PS1 mice and WT mice. Data are presented as means ± SD (*n* = 3; *p < 0.05).

Through the predictive analysis of miRNA-mRNA interactions, we identified the downstream target mRNAs that might be regulated and constructed a circRNA-ceRNA network related to the three signaling pathways (Figure 5A and Table 5). Based on the regulatory mechanism of circRNA-ceRNA, we ultimately screened the novel_circ_0003012/mmu-miR-298-3p/Smoc2 signaling axis, which might affect the cGMP-PKG signaling pathway (Figure 5B). We used qPCR to verify the differential expression of these genes, and used WB to verify the level of Smoc2, the results are shown in Figures 5C,D.

**TABLE 5:** CircRNA-ceRNA networks in cGMP-PKG, cAMP, and Hippo signaling pathway.

**CircRNA name**	**MiRNA name**	**Gene_id**	**Gene name**
novel_circ_0009273	mmu-miR-3074-1-3p	ENSMUSG00000031486	Adgra2
novel_circ_0003012	mmu-miR-298-3p	ENSMUSG00000023886	Smoc2
novel_circ_0006299	mmu-miR-296-3p	ENSMUSG00000026825	Dnm1
novel_circ_0006299	mmu-miR-296-3p	ENSMUSG00000046854	Pip5kl1
novel_circ_0006299	mmu-miR-296-3p	ENSMUSG00000004789	Dlst
novel_circ_0006299	mmu-miR-296-3p	ENSMUSG00000023886	Smoc2
novel_circ_0006299	mmu-miR-296-3p	ENSMUSG00000024969	Mark2
novel_circ_0006299	mmu-miR-296-3p	ENSMUSG00000042632	Pla2g6
novel_circ_0008567	mmu-miR-298-5p	ENSMUSG00000028931	Kcnab2
novel_circ_0008567	mmu-miR-298-5p	ENSMUSG00000024942	Capn1
novel_circ_0008567	mmu-miR-298-5p	ENSMUSG00000031169	Porcn
novel_circ_0008567	mmu-miR-298-5p	ENSMUSG00000020532	Acaca
novel_circ_0008567	mmu-miR-298-5p	ENSMUSG00000028801	Stpg1
novel_circ_0008567	mmu-miR-298-5p	ENSMUSG00000005936	Kctd20
novel_circ_0008567	mmu-miR-298-5p	ENSMUSG00000023886	Smoc2
novel_circ_0008567	mmu-miR-298-5p	ENSMUSG00000022377	Asap1
novel_circ_0008567	mmu-miR-298-5p	ENSMUSG00000015766	Eps8
mmu_circ_0000585	mmu-miR-3074-1-3p	ENSMUSG00000031486	Adgra2

Furthermore, we also verified whether the circRNA-ceRNA network affects the cGMP-PKG signaling pathway. As shown in Figure 5E, we used Western blotting to detect the expression of PKG, a key factor in the cGMP-PKG signaling pathway, and the results showed that the expression of PKG in the hippocampus of APP/PS1 mice was significantly reduced compared with that in the WT group (*p* < 0.05).

Preliminary verification of the regulatory role of the novel_circ_0003012/mmu-miR-298-3p/Smoc2 signaling axis in the pathology of AD showed that it involved the downregulation of PKG.

## Discussion

Analyzing the expression profiles of circRNA-ceRNA may provide new insights into our understanding of the pathophysiology of AD. In our study, we found 70 dysregulated circRNAs, 39 dysregulated miRNAs and 121 dysregulated mRNA between the APP/PS1 group and wild-type group at 8 months in the hippocampus of the mice; 44 circRNAs, 16 miRNAs and 34 mRNAs were upregulated, and 26 circRNAs, 23 miRNAs, and 87 mRNAs were downregulated in APP/PS1 mice relative to their levels in wild-type mice. Through correlation analysis, we obtained 11 dysregulated circRNAs (7 upregulated circRNAs and 4 downregulated), 7 dysregulated miRNAs (4 upregulated miRNAs and 3 downregulated) and 8 dysregulated mRNAs (3 upregulated mRNAs and 5 downregulated), forming 16 relationships in the circRNA-miRNA-mRNA regulatory network. Our results showed that the aberrantly expressed circRNAs had miRNA-binding sites and were thus predicted to play a regulatory role via the ceRNA mechanism (Zhang et al., 2020). These circRNA-miRNA-mRNA regulatory networks may play an important role in the onset and development of AD. For instance, mmu_circ_0001125 and mmu_circ_0000672 were found to be ceRNAs of mmu-miR-351-5p, which targets *Cst7* (ENSMUSG00000068129). Cst7 (cystatin F) encodes an endosomal/lysosomal cathepsin inhibitor that regulates cathepsin activity in the lysosomal pathway (Magister and Kos, 2013). The expression of Cst7 is important in microglia for reducing the phagocytic capacity of activated microglia. Reducing the expression of Cst7 might promote the clearance of Aβ species through microglia and macrophages (Ofengeim et al., 2017).

The GO analysis was performed to further annotate the biological functions related to the aberrantly expressed circRNAs. The top GO terms of the differentially expressed circRNAs were most enriched in biological metabolic processes, such as phosphorus metabolic process, organophosphate metabolic process, nucleotide metabolic process, nucleoside phosphate metabolic process, pyrimidine nucleobase metabolic process, ribonucleotide metabolic process and ribose phosphate metabolic process. This result indicated that the pathological progression of AD may be associated with several metabolic pathways regulated by circRNAs. In addition, we also performed GO analysis of miRNAs and mRNAs. The top terms of miRNAs were almost all associated with cellular metabolic process, intracellular organelle/part and binding functions. The dysregulated mRNAs were mostly enriched in cellular/organism development processes or cell differentiation.

As the main components of nucleic acids, nucleobases, nucleosides, nucleotides and related phosphorylated metabolites have many important roles as intermediates in biosynthetic pathways in biological systems (Lane and Fan, 2015; Swerdlow, 2018; Muguruma et al., 2020). There is growing evidence that nucleotide metabolism is involved in pathological mechanisms in many different neurodegenerative diseases, such as Alzheimer’s disease. As Gonzalez-Dominguez et al. (2015) revealed, numerous metabolites, including purine and pyrimidine metabolites, show significant differences between AD and WT mice in all brain tissues, especially in hippocampal and cortical regions. This result indicated that alterations in the metabolism of nucleotides play an important role in the pathological process of AD. For instance, purinergic signaling plays a critical role in the development of AD. Studies have demonstrated that adenosine receptors in the frontal cortex of the affected brain are upregulated and that these receptors are redistributed. Furthermore, these receptors have higher activity in neurons affected by Aβ deposition (Albasanz et al., 2008; Cieslak and Wojtczak, 2018).

In addition, abnormal synthesis or metabolism of pyrimidine nucleotides is also considered to be an important factor in the pathological process of AD, and its disorder can cause dysfunction of the oxidative phosphorylation (OXPHOS) system (Pesini et al., 2019). The OXPHOS system plays an important role in the mechanism of synaptic failure and neurodegeneration triggered by Aβ (Pesini et al., 2014). After Aβ deposition, OXPHOS dysfunction appears to be a frequent finding in many AD patients (Swerdlow et al., 2014). OXPHOS participates in many cellular processes, and defects in this system affect many biochemical pathways. One of these biochemical pathways is *de novo* pyrimidine biosynthesis. A decrease in the *de novo* synthesis of pyrimidine nucleotides leads to dysfunction of the OXPHOS system and to the pathogenesis of late-onset AD (Pesini et al., 2019). Disorders of pyrimidine metabolism, with decreased uridine monophosphate and increased uracil, ultimately lead to synaptic plasticity and neuronal impairment (Czech et al., 2012). Several studies have also indicated that oxidative stress is closely related to the abnormal metabolism of purines and pyrimidines in AD (Lyras et al., 1997; Gonzalez-Dominguez et al., 2015). All these results indicate that abnormal nucleotide metabolism is also an important factor in the onset and development of AD. These circRNAs in the hippocampus of AD mice may play a critical role in the pathological progression of AD by regulating nucleotide metabolism.

Kyoto Encyclopedia of Genes and Genomes analysis showed that the dysregulation of circRNAs was enriched in many signaling pathways, which are closely related to the pathogenesis of AD, including the cGMP-PKG signaling pathway, cAMP signaling pathway, Hippo signaling pathway, platelet activation, LTP and axon guidance. KEGG pathway analysis of dysregulated miRNAs and mRNAs also identified enrichment in focal adhesion, axon guidance, platelet activation and the Hippo signaling pathway. In particular, the signaling pathways of miRNA and mRNA enrichment, such as platelet activation, axon guidance and the Hippo signaling pathway, were consistent with the KEGG analysis of circRNAs.

Other studies have also reported that several dysregulated circRNAs in the cerebral cortex of AD mice are enriched in the PI3K-Akt signaling pathway, tight junctions, Hippo signaling pathway, LTP and axon guidance (Zhang et al., 2017; Ma et al., 2019). These results are consistent with our study that the dysregulated circRNAs in the hippocampus of AD mice were also enriched in the Hippo signaling pathway, LTP and axon guidance. The Hippo signaling pathway, also known as the Salvador/Warts/Hippo (SWH) pathway, is named after the protein kinase Hippo (Hpo) in Drosophila and is a key regulator in the pathway (Zheng and Pan, 2019). The Hippo pathway is composed of a series of conserved kinases (Huang et al., 2005). Numerous studies have confirmed that the Hippo signaling pathway plays an important role in cell functions. Hippo signaling activates induced cell death, whereas inactivation of Hippo signaling triggers cell proliferation (Grusche et al., 2011; Sun and Irvine, 2011). A recent study indicated that the Hippo pathway plays an important role in the pathogenesis of AD. The Hippo pathway affects Aβ42-mediated neurodegeneration due to the excessive activation of Hippo signaling, leading to enhanced Aβ42 toxicity; however, downregulation of the Hippo signaling pathway can rescue Aβ42-mediated neurodegeneration (Irwin et al., 2020).

Interestingly, we also found that the signaling pathways of the cGMP-PKG signaling pathway, cAMP signaling pathway and platelet activation in the hippocampus of AD mice were associated with dysregulated circRNAs in the pathogenesis of AD (Kelly, 2018; Ricciarelli and Fedele, 2018). Cyclic adenosine monophosphate (cAMP) and cyclic guanosine monophosphate (cGMP) are well-established second messengers required for LTP and memory formation and consolidation (Ricciarelli and Fedele, 2018). Recent evidence indicates that excessive Aβ deposition inhibits both the cAMP and cGMP pathways and impairs LTP signal transduction. Changes in cAMP signals in specific brain regions may be related to the pathology of dementia. Reduced cAMP signaling is an important factor in AD pathology. Increasing cAMP signaling in specific regions of the brain can resist age-related declines in brain function. Studies have shown that cAMP levels in the hippocampus can be reduced by the overexpression of β-site amyloid precursor protein-cleaving enzyme 1 (BACE1) or the infusion of Aβ_1__–__42_ (Chen et al., 2012; Zhang et al., 2014). Furthermore, cAMP-elevating agents can reverse or prevent Aβ-induced hippocampal deficits.

Cyclic guanosine monophosphate-dependent protein kinase (PKG) and the cGMP controller phosphodiesterase are critical participants in the neuroinflammatory process, which may lead to neurological dysfunction, cell death and further neurodegeneration (Ricciarelli and Fedele, 2018). The increase in cGMP levels decreases the Aβ load in transgenic models of AD and in models of physiological aging (Sierksma et al., 2013). In addition, cGMP-dependent Akt activation and GSK3β inactivation can reduce tau hyperphosphorylation. PKG, as the key downstream target of cGMP, has been reported to be significantly decreased in both the cortex and hippocampus after treatment with Aβ (Wang et al., 2017). The cGMP-PKG pathway plays a crucial role in preventing apoptosis and promoting neural cell survival. It has been shown that the activation of PKG in hippocampal neurons is involved in the LTP induced by NO and carbon monoxide (Fiscus, 2002). Inhibition of PKG activity in hippocampal neurons can partially block the prosurvival effects of APP^S^, suggesting that cGMP, via activation of PKG, mediates the neuroprotective effect of APP^S^ (Barger et al., 1995; Fiscus, 2002). Our results are consistent with the above research, confirming that the expression of PKG in the hippocampus was obviously decreased in AD mice and that the cGMP-PKG pathway might play an essential role in the pathophysiology of AD.

From the results of the circRNA-ceRNA network constructed on the basis of the differentially expressed circRNAs, miRNAs and mRNAs obtained from the sequencing analysis results and the series of circRNAs predicted by KEGG analysis that are closely related to the cGPM-PKG signaling pathway, we found that the novel_circ_0003012/mmu-miR-298-3p/Smoc2 signaling axis may be closely related to the pathological mechanism of AD.

Through preliminary verification, we found that the differential expression of novel_circ_0003012 and mmu-miR-298-3p may regulate the pathological mechanism of AD by affecting the cGPM-PKG signaling pathway.

## Conclusion

In summary, we elucidated the circRNA-ceRNA network patterns in the hippocampus of APP/PS1 and WT mice by using deep RNA-seq analysis. Our findings further expand the current knowledge regarding the biology of circRNA-ceRNA, their involved signaling pathways, such as the dysregulated circRNAs in nucleotide metabolism, cGMP-PKG signaling pathway, cAMP signaling pathway, platelet activation and Hippo signaling pathway, and their regulatory roles in AD pathogenesis. In addition, our findings preliminarily verified that the novel_circ_0003012/mmu-miR-298-3p/Smoc2 signaling axis may regulate the pathophysiology of AD by affecting the cGMP-PKG signaling pathway. These newly identified circRNAs in networks and signaling pathways reveal potential diagnostic or therapeutic targets for AD.

## Data Availability Statement

The datasets presented in this study can be found in online repositories. The names of the repository/repositories and accession number(s) can be found below: https://www.ncbi.nlm.nih.gov/sra/?term=PRJNA712946.

## Ethics Statement

The animal study was reviewed and approved by Medical Ethics Committee of Qingdao University.

## Author Contributions

YZ conceived and wrote the manuscript. YZ and LQ reviewed and edited the manuscript. YLy, YLg, WY, and YZf participatedin literature search, data collection, and figures design. All authors read and approved the manuscript.

## Conflict of Interest

The authors declare that the research was conducted in the absence of any commercial or financial relationships that could be construed as a potential conflict of interest.

## Footnotes

1 http://www.genome.jp/kegg/

2 www.targetscan.org

3 www.microrna.org/microrna/home.do

4 http://ccb.jhu.edu/software/hisat2

## Abbreviations

AD: Alzheimer’s disease
BACE1: β-site amyloid precursor protein-cleaving enzyme 1
BP: biological process
cAMP: cyclic adenosine monophosphate
CC: cellular component
ceRNAs: competitive endogenous RNAs
circRNAs: circular RNAs
cGMP: cyclic guanosine monophosphate
Cst7: cystatin F
GO: Gene ontology
KEGG: Kyoto Encyclopedia of Genes and Genomes
LTP: long-term potentiation
MF: molecular function
MREs: miRNA response elements
OXPHOS: oxidative phosphorylation
PKG: cGMP-dependent protein kinase
WT: wild-type.
