# Supplementary material for: CircRNA-ceRNA Network Revealing the Potential Regulatory Roles of CircRNA in Alzheimer’s Disease Involved the cGMP-PKG Signal Pathway
Source: Front Mol Neurosci. 2021 May 21;14:665788. doi: 10.3389/fnmol.2021.665788 (PMC8176118; doi:10.3389/fnmol.2021.665788)
Supplement: Supplementary file 1 [file Data_Sheet_1.DOCX]

**CircRNA Methods**

**Library preparation for Transcriptome sequencing**

A total amount of 3 µg RNA per sample was used as input material for the RNA sample preparations. Sequencing libraries were generated using NEBNext® UltraTM RNA Library Prep Kit for Illumina® (NEB, USA) following manufacturer’s recommendations and index codes were added to attribute sequences to each sample. Briefly, mRNA was purified from total RNA using poly-T oligo-attached magnetic beads. Fragmentation was carried out using divalent cations under elevated temperature in NEBNext First Strand Synthesis Reaction Buffer(5X). First strand cDNA was synthesized using random hexamer primer and M-MuLV Reverse Transcriptase (RNase H-). Second strand cDNA synthesis was subsequently performed using DNA Polymerase I and RNase H-. Remaining overhangs were converted into blunt ends via exonuclease/polymerase activities. After adenylation of 3’ ends of DNA fragments, NEBNext Adaptor with hairpin loop structure were ligated to prepare for hybridization. In order to select cDNA fragments of preferentially 150~200 bp in length, the library fragments were purified with AMPure XP system (Beckman Coulter, Beverly, USA). Then 3 µl USER Enzyme (NEB, USA) was used with size-selected, adaptor-ligated cDNA at 37°C for 15 min followed by 5 min at 95 °C before PCR. Then PCR was performed with Phusion High-Fidelity DNA polymerase, Universal PCR primers and Index (X) Primer. At last, PCR products were purified (AMPure XP system) and library quality was assessed on the Agilent Bioanalyzer 2100 system.

**Clustering and sequencing**

The clustering of the index-coded samples was performed on a cBot Cluster Generation System using TruSeq PE Cluster Kit v3-cBot-HS (Illumia) according to the manufacturer’s instructions. After cluster generation, the library preparations were sequenced on an Illumina Hiseq platform and 125 bp/150 bp paired-end reads were generated.

**Data Analysis**

**Quality control**

Raw data (raw reads) of fastq format were firstly processed through in-house perl scripts. In this step, clean data (clean reads) were obtained by removing reads containing adapter, reads containing ploy-N and low quality reads from raw data. At the same time, Q20, Q30 and GC content the clean data were calculated. All the downstream analyses were based on the clean data with high quality.

**Reads mapping to the reference genome**

Reference genome and gene model annotation files were downloaded from genome website directly. Index of the reference genome was built using STAR and paired-end clean reads were aligned to the reference genome using STAR (v2.5.1b). STAR used the method of Maximal Mappable Prefix (MMP) which can generate a precise mapping result for junction reads.

**circRNA identification**

The circRNA were detected and identified using find_circ (Sebastian Memczak et al., 2013) and CIRI2 (Gao et al., 2017). Circos software was used to construct the circos figure.

**Quantification of gene expression level**

HTSeq v0.6.0 was used to count the reads numbers mapped to each gene. And then FPKM of each gene was calculated based on the length of the gene and reads count mapped to this gene. FPKM, expected number of Fragments Per Kilobase of transcript sequence per Millions base pairs sequenced, considers the effect of sequencing depth and gene length for the reads count at the same time, and is currently the most commonly used method for estimating gene expression levels.

**Differential expression analysis**

(For DESeq2 with biological replicates) Differential expression analysis of two conditions/groups (two biological replicates per condition) was performed using the DESeq2 R package (1.10.1). DESeq2 provide statistical routines for determining differential expression in digital gene expression data using a model based on the negative binomial distribution. The resulting P-values were adjusted using the Benjamini and Hochberg’s approach for controlling the false discovery rate. Genes with an adjusted P-value <0.05 found by DESeq2 were assigned as differentially expressed.

(For edgeR without biological replicates) Prior to differential gene expression analysis, for each sequenced library, the read counts were adjusted by edgeR program package through one scaling normalized factor. Differential expression analysis of two conditions was performed using the edgeR R package (3.12.1). The P values were adjusted using the Benjamini & Hochberg method. Corrected P-value of 0.05 and absolute fold change of 2 were set as the threshold for significantly differential expression.

**MicroRNA target site analysis**

MicroRNA target site in exons of circRNA loci were identified using miRanda (animal species).

**CircRNA-miRNA-gene network analysis**

Cytoscape software was used to construct the circRNA-miRNA-gene networks.

**microRNA Methods**

**RNA isolation, quantification and qualification**

RNA degradation and contamination was monitored on 1% agarose gels. RNA purity was checked using the NanoPhotometer® spectrophotometer (IMPLEN, CA, USA). RNA concentration was measured using Qubit® RNA Assay Kit in Qubit® 2.0 Flurometer (Life Technologies, CA, USA). RNA integrity was assessed using the RNA Nano 6000 Assay Kit of the Agilent Bioanalyzer 2100 system (Agilent Technologies, CA, USA).

**Library preparation for Small RNA sequencing**

A total amount of 3 μg total RNA per sample was used as input material for the small RNA library. Sequencing libraries were generated using NEBNext® Multiplex Small RNA Library Prep Set for Illumina® (NEB, USA.) following manufacturer’s recommendations and index codes were added to attribute sequences to each sample. Briefly, NEB 3' SR Adaptor was directly, and specifically ligated to 3' end of miRNA, siRNA and piRNA. After the 3' ligation reaction, the SR RT Primer hybridized to the excess of 3' SR Adaptor (that remained free after the 3' ligation reaction) and transformed the single-stranded DNA adaptor into a double-stranded DNA molecule. This step is important to prevent adaptor-dimer formation, besides, dsDNAs are not substrates for ligation mediated by T4 RNA Ligase 1 and therefore do not ligate to the 5´SR Adaptor in the subsequent ligation step. 5´ends adapter was ligated to 5´ends of miRNAs, siRNA and piRNA. Then first strand cDNA was synthesized using M-MuLV Reverse Transcriptase (RNase H-). PCR amplification was performed using LongAmp Taq 2X Master Mix, SR Primer for illumine and index (X) primer. PCR products were purified on a 8% polyacrylamide gel (100V, 80 min). DNA fragments corresponding to 140~160 bp (the length of small noncoding RNA plus the 3' and 5' adaptors) were recovered and dissolved in 8 μL elution buffer. At last, library quality was assessed on the Agilent Bioanalyzer 2100 system using DNA High Sensitivity Chips.

**Clustering and sequencing**

The clustering of the index-coded samples was performed on a cBot Cluster Generation System using TruSeq SR Cluster Kit v3-cBot-HS (Illumia) according to the manufacturer’s instructions. After cluster generation, the library preparations were sequenced on an Illumina Hiseq 2500/2000 platform and 50bp single-end reads were generated.

**Data analysis**

**Quality control**

Raw data (raw reads) of fastq format were firstly processed through custom perl and python scripts. In this step, clean datas (clean reads) were obtained by removing reads containing ploy-N, with 5’ adapter contaminants, without 3’ adapter or the insert tag, containing ploy A or T or G or C and low quality reads from raw data. At the same time, Q20, Q30, and GC-content of the raw datas were calculated. Then, chose a certain range of length from clean reads to do all the downstream analyses.

**Reads mapping to the reference sequence**

The small RNA tags were mapped to reference sequence by Bowtie (Langmead et al., 2009) without mismatch to analyze their expression and distribution on the reference.

**Known miRNA alignment**

Mapped small RNA tags were used to looking for known miRNA. miRBase20.0 was used as reference, modified software mirdeep2 (Friedlander et al., 2011) and srna-tools-cli were used to obtain the potential miRNA and draw the secondary structures. Custom scripts were used to obtain the miRNA counts as well as base bias on the first position of identified miRNA with certain length and on each position of all identified miRNA respectively.

**Remove tags from these sources**

To remove tags originating from protein-coding genes, repeat sequences, rRNA, tRNA, snRNA, and snoRNA, small RNA tags were mapped to RepeatMasker, Rfam database or those types of datas from the specified species itself.

**Novel miRNA prediction**

The characteristics of hairpin structure of miRNA precursor can be used to predict novel miRNA. The available software miREvo (Wen et al., 2012) and mirdeep2 (Friedlander et al., 2011) were integrated to predict novel miRNA through exploring the secondary structure, the Dicer cleavage site and the minimum free energy of the small RNA tags unannotated in the former steps. At the same time, custom scripts were used to obtain the identified miRNA counts as well as base bias on the first position with certain length and on each position of all identified miRNA respectively.

**Small RNA annotation summary**

Summarizing all alignments and annotations obtained before. In the alignment and annotation before, some small RNA tags may be mapped to more than one category. To make every unique small RNA mapped to only one annotation, we follow the following priority rule: known miRNA > rRNA > tRNA > snRNA > snoRNA > repeat > gene > NAT-siRNA > gene > novel miRNA > ta-siRNA. The total rRNA proportion was used a marker as sample quality indicator. Usually it should be less than 60% in plant samples and 40% in animal samples as high quality.

**miRNA editing analysis**

Position 2~8 of a mature miRNA were called seed region which were highly conserved. The target of a miRNA might be different with the changing of nucleotides in this region. In our analysis pipeline, miRNA which might have base edit could be detected by aligning all the sRNA tags to mature miRNA, allowing one mismatch.

**miRNA family analysis**

Exploring the occurrence of miRNA families identified from the samples in other species. In our analysis pipeline, known miRNA used miFam.dat (http://www.mirbase.org/ftp.shtml) to look for families; novel miRNA precursor was submitted to Rfam (<http://rfam.sanger.ac.uk/search/>) to look for Rfam families.

**Target gene prediction**

Predicting the target gene of miRNA was performed by miRanda (Enright et al, 2003) for animals.

**Quantification of miRNA**

miRNA expression levels were estimated by TPM (transcript per million) through the following criteria (Zhou et al., 2010): Normalization formula: Normalized expression = mapped readcount/Total reads*1000000.

**Differential expression of miRNA**

For the samples with biological replicates:

Differential expression analysis of two conditions/groups was performed using the DESeq R package (1.8.3). The P-values was adjusted using the Benjamini & Hochberg method. Corrected P-value of 0.05 was set as the threshold for significantly differential expression by default.

For the samples without biological replicates:

Differential expression analysis of two samples was performed using the DEGseq (2010) R package. P-value was adjusted using qvalue (Storey et al, 2003). qvalue<0.01 and |log2(fold change)|>1 was set as the threshold for significantly differential expression by default.

**GO and KEGG enrichment analysis**

Gene Ontology (GO) enrichment analysis was used on the target gene candidates of differentially expressed miRNAs (“target gene candidates” in the following). GOseq based Wallenius non-central hyper-geometric distribution (Young et al, 2010), which could adjust for gene length bias, was implemented for GO enrichment analysis.

KEGG (Kanehisa et al., 2008) is a database resource for understanding high-level functions and utilities of the biological system, such as the cell, the organism and the ecosystem, from molecular-level information, especially large-scale molecular datasets generated by genome sequencing and other high-throughput experimental technologies (<http://www.genome.jp/kegg/>). We used KOBAS (Mao et al., 2005) software to test the statistical enrichment of the target gene candidates in KEGG pathways.

**mRNA Methods**

**RNA quantification and qualification**

RNA degradation and contamination was monitored on 1% agarose gels. RNA purity was checked using the NanoPhotometer® spectrophotometer (IMPLEN, CA, USA).

RNA integrity was assessed using the RNA Nano 6000 Assay Kit of the Bioanalyzer 2100 system (Agilent Technologies, CA, USA).

**Library preparation for Transcriptome sequencing**

A total amount of 1 µg RNA per sample was used as input material for the RNA sample preparations. Briefly, mRNA was purified from total RNA using poly-T oligo-attached magnetic beads. Fragmentation was carried out using divalent cations under elevated temperature in First Strand Synthesis Reaction Buffer. First strand cDNA was synthesized using random hexamer primer and RNase H. In order to select cDNA fragments of preferentially 100~200 bp in length, the library fragments were purified with AMPure XP system (Beckman Coulter, Beverly, USA).

Adapter ligation at 25°C for 10min before PCR. Then PCR was performed with Phusion High-Fidelity DNA polymerase, Universal PCR primers and Index (X) Primer. At last, PCR products were purified (AMPure XP system) and library quality was assessed on the Agilent Bioanalyzer 2100 system.

**Clustering and sequencing**

The clustering of the index-coded samples was performed on a cBot Cluster Generation System using TruSeq PE Cluster Kit v3-cBot-HS (Illumia) according to the manufacturer’s instructions. After cluster generation, the library preparations were sequenced on an Illumina platform and 150 bp paired-end reads were generated.

**Data analysis**

**Quality control**

Raw data (raw reads) of fastq format were firstly processed through in-house perl scripts. In this step, clean data (clean reads) were obtained by removing reads containing adapter, reads containing ploy-N and low quality reads from raw data. At the same time, Q20, Q30 and GC content of the clean data were calculated. The UMI (Unique Molecular Identifiers) was extracted by UMI-tools v1.0.0. All the downstream analyses were based on the clean UMI reads with high quality.

**Mapping reads to reference genome and deduplicating reads by UMI mapping coordinates**

Reference genome and gene model annotation files were downloaded from genome website directly. Index of the reference genome was built using Hisat2 v2.0.4 and paired- end clean reads were aligned to the reference genome using Hisat2 v2.0.4. We selected Hisat2 as the mapping tool for that Hisat2 can generate a database of splice junctions based on the gene model annotation file and thus a better mapping result than other non-splice mapping tools. We used UMI-tools v1.0.0 to deduplicate reads based on the mapping co- ordinate and the UMI attached to the read.

**Quantification of gene expression level**

HTSeq v0.9.1 was used to count the reads numbers mapped to each gene. And then FPKM of each gene was calculated based on the length of the gene and reads count mapped to this gene. FPKM, expected number of Fragments Per Kilobase of transcript sequence per Millions base pairs sequenced, considers the effect of sequencing depth and gene length for the reads count at the same time, and is currently the most commonly used method for estimating gene expression levels (Trapnell, Cole, et al., 2010).

**Differential expression analysis**

(For DESeq with biological replicates) Differential expression analysis of two conditions/groups (two biological replicates per condition) was performed using the DESeq R package (1.18.0). DESeq provide statistical routines for determining differential expression in digital gene expression data using a model based on the negative binomial distribution.

The resulting P-values were adjusted using the Benjamini and Hochberg’s approach for controlling the false discovery rate. Genes with an adjusted P-value <0.05 found by DESeq were assigned as differentially expressed.

(For DEGSeq without biological replicates) Prior to differential gene expression analysis, for each sequenced library, the read counts were adjusted by edgeR program package through one scaling normalized factor.

Differential expression analysis of two conditions was performed using the DEGSeq R package (1.20.0). The P values were adjusted using the Benjamini & Hochberg method. Corrected P-value of 0.005 and log2(Fold change) of 1 were set as the threshold for significantly differential expression.

**GO and KEGG enrichment analysis of differentially expressed genes**

Gene Ontology (GO) enrichment analysis of differentially expressed genes was implemented by the GOseq R package, in which gene length bias was corrected. GO terms with corrected P-value less than 0.05 were considered significantly enriched by differential expressed genes.

KEGG is a database resource for understanding high-level functions and utilities of the biological system, such as the cell, the organism and the ecosystem, from molecular-level information, especially large-scale molecular datasets generated by genome sequencing and other high-through put experimental technologies (<http://www.genome.jp/kegg/>). We used KOBAS software to test the statistical enrichment of differential expression genes in KEGG pathways.

**PPI analysis of differentially expressed genes**

PPI analysis of differentially expressed genes was based on the STRING database, which known and predicted Protein-Protein Interactions. For the species existing in the database, we construct the networks by extract the target gene list from the database; Otherwise, Blastx (v2.2.28) was used to align the target gene sequences to the selected reference protein sequences, and then the networks was built according to the known interaction of selected reference species.

**Novel transcripts prediction and alternative splicing analysis**

The Cufflinks v2.1.1 Reference Annotation Based Transcript (RABT) assembly method was used to construct and identify both known and novel transcripts from Hisat2 alignment results. Alternative splicing events were classified to 5 basic types by the software rMATS v3.2.5. The number of AS events in each compare group was estimated, separately.

**Real-time amplification curve and melt curve analysis for gene expression**

Real-time amplification curve and melt curve analysis for mmu_circ_0000672 between 8-month-old APP/PS1 and WT mice

**
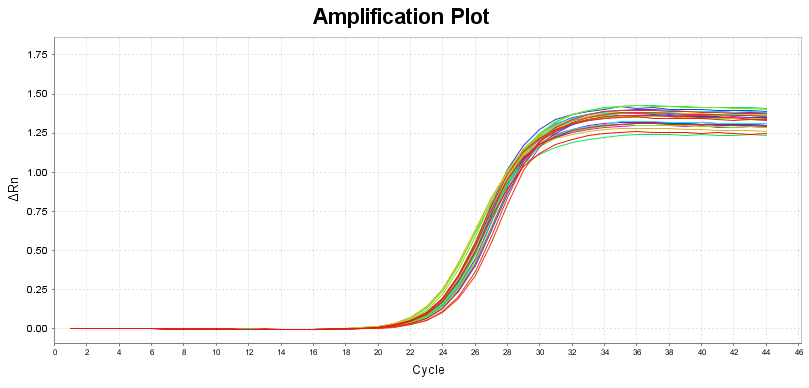

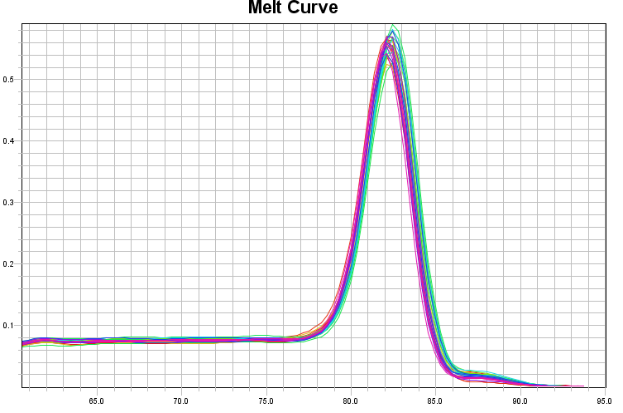
**

Real-time amplification curve and melt curve analysis for novel_circ_0001016 between 8-month-old APP/PS1 and WT mice

**
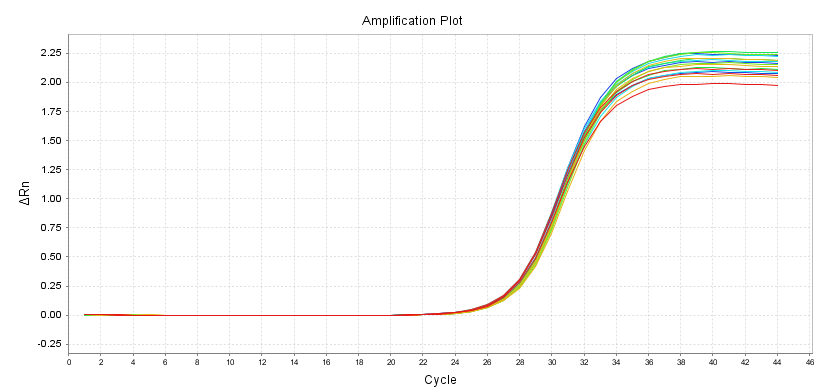

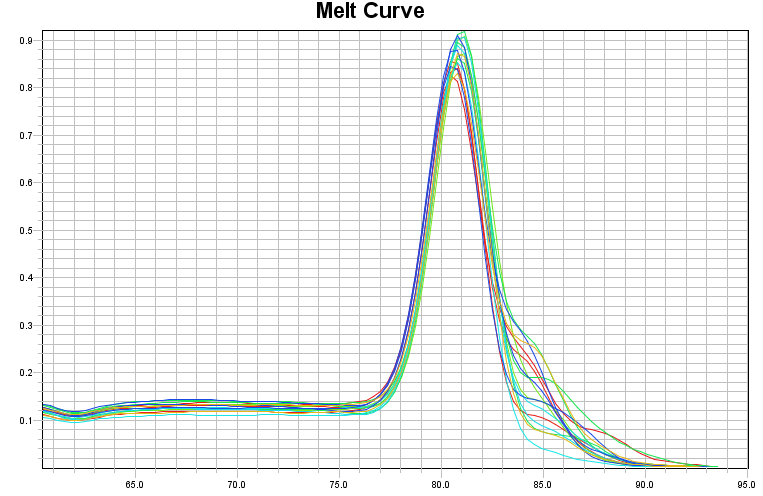
**

Real-time amplification curve and melt curve analysis for novel_circ_0005255 between 8-month-old APP/PS1 and WT mice

**
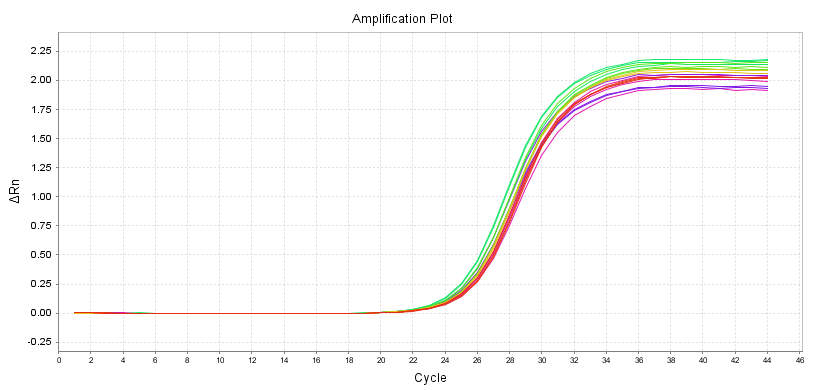

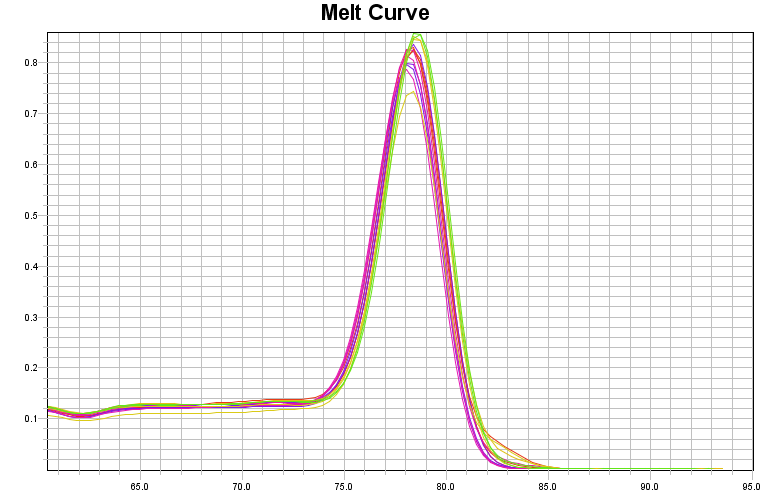
**

Real-time amplification curve and melt curve analysis for mmu-miR-344d-3-5p between 8-month-old APP/PS1 and WT mice

**
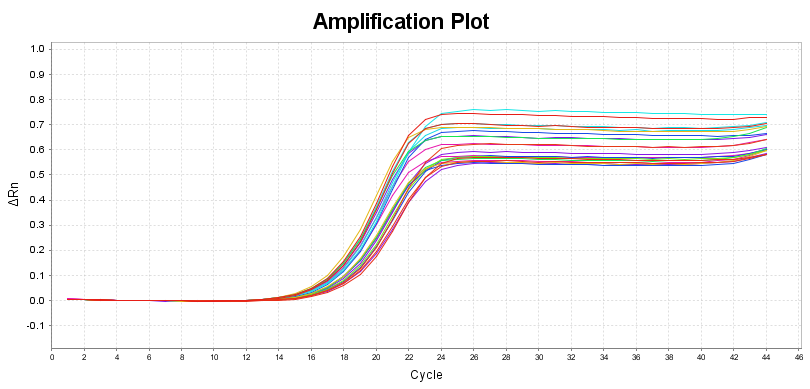

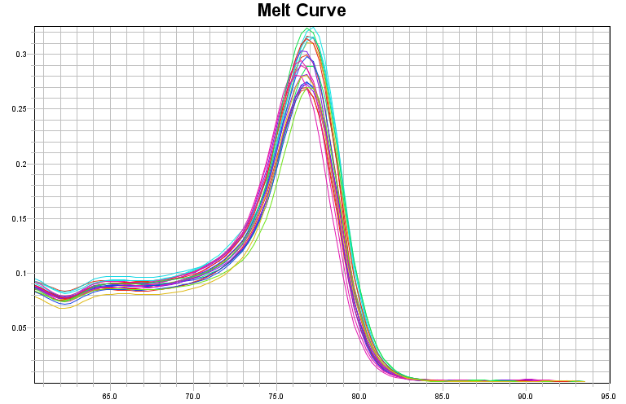
**

Real-time amplification curve and melt curve analysis for mmu-miR-296-3p between 8-month-old APP/PS1 and WT mice

**
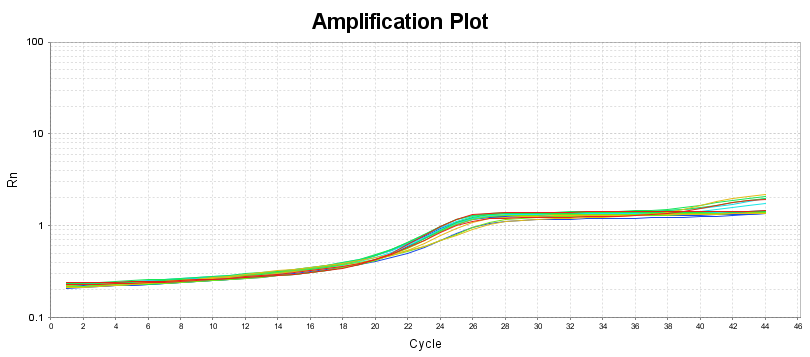

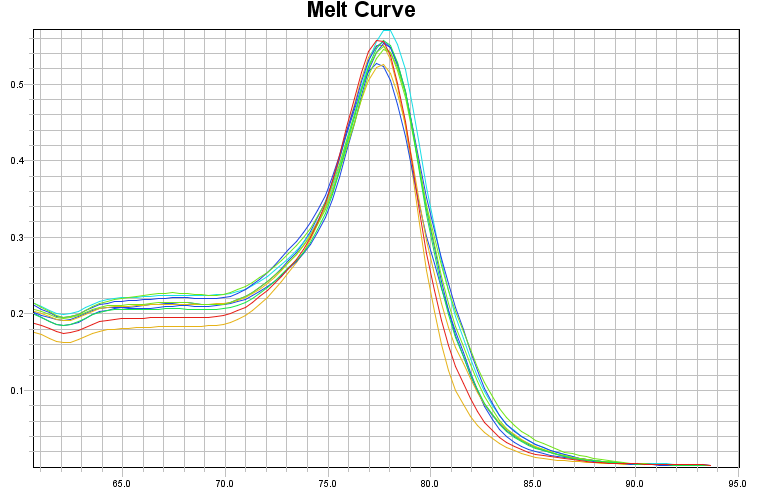
**

Real-time amplification curve and melt curve analysis for mmu-miR-222-3p between 8-month-old APP/PS1 and WT mice

**
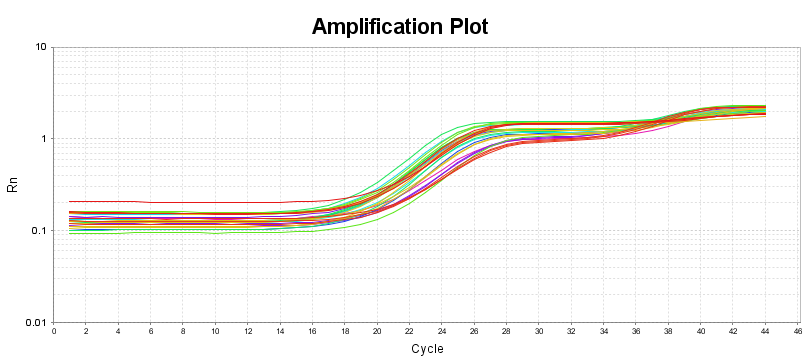

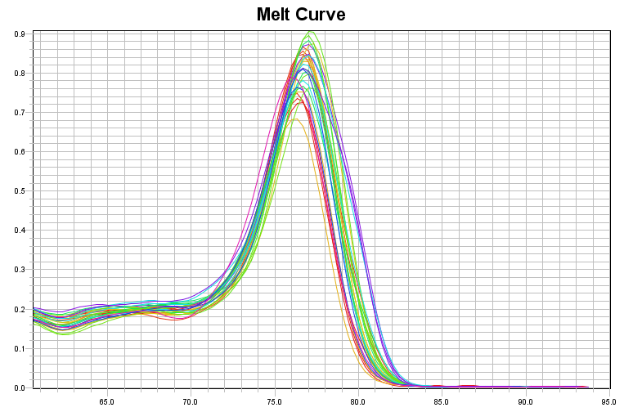
**

Real-time amplification curve and melt curve analysis for mmu-miR-298-3p between 8-month-old APP/PS1 and WT mice

**
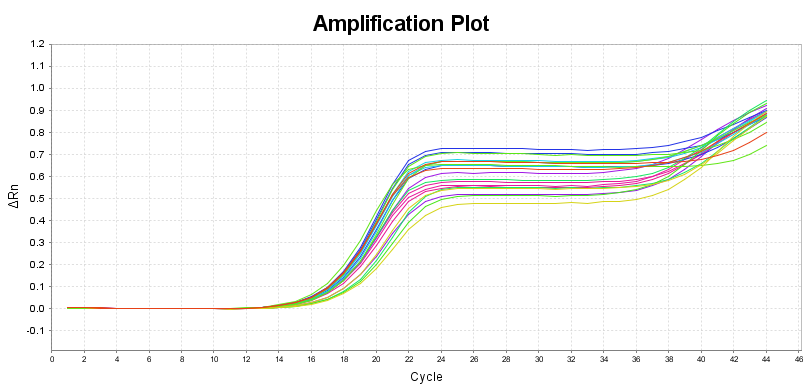

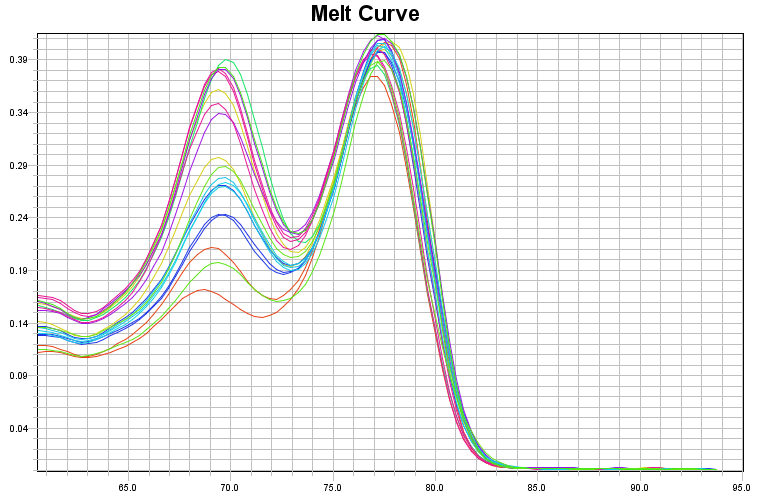
**

Real-time amplification curve and melt curve analysis for ENSMUSG00000068129 between 8-month-old APP/PS1 and WT mice

**
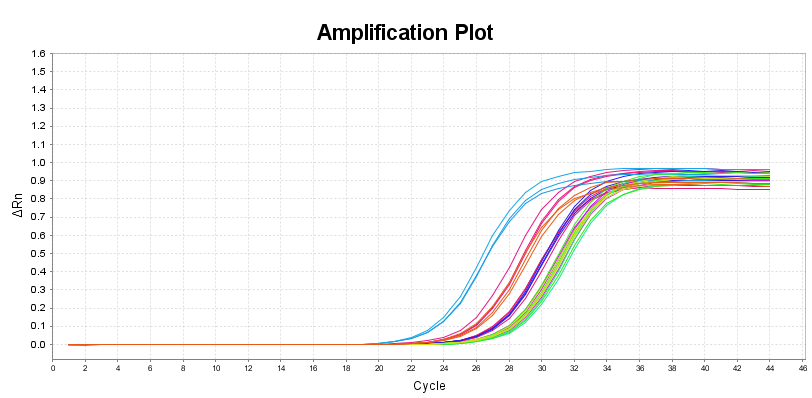

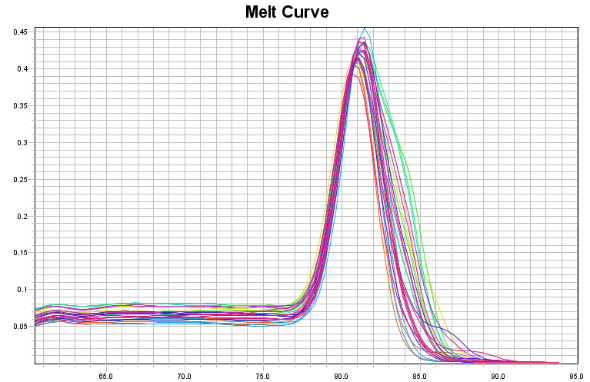
**

Real-time amplification curve and melt curve analysis for ENSMUSG00000006179 between 8-month-old APP/PS1 and WT mice

**
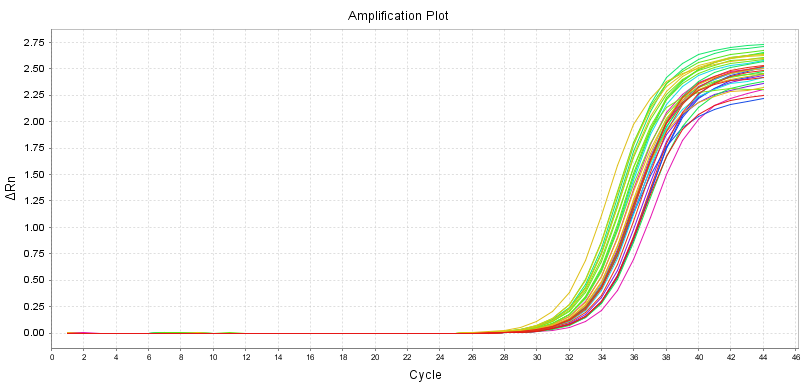

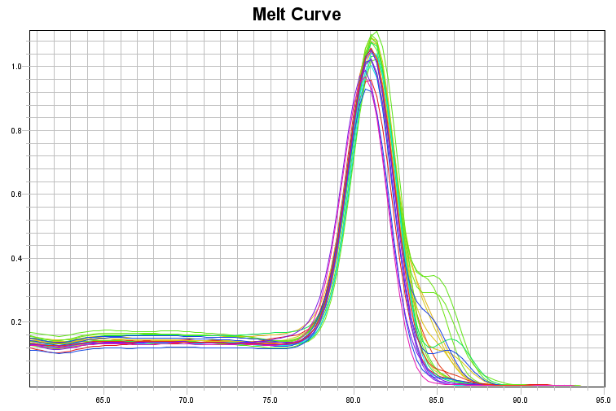
**

Real-time amplification curve and melt curve analysis for ENSMUSG00000040298 between 8-month-old APP/PS1 and WT mice

**
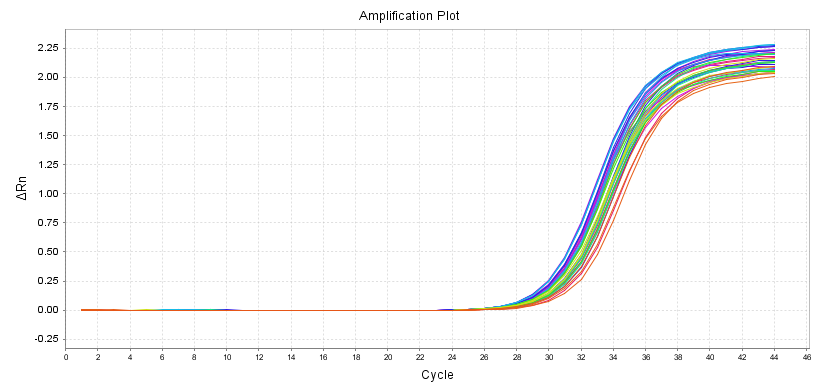

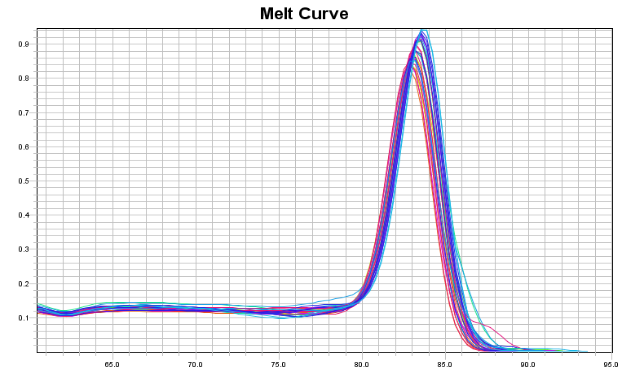
**

Real-time amplification curve and melt curve analysis for GAPDH

**
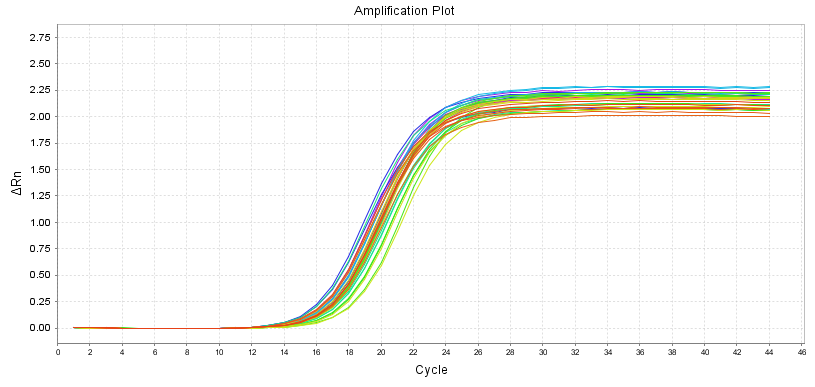

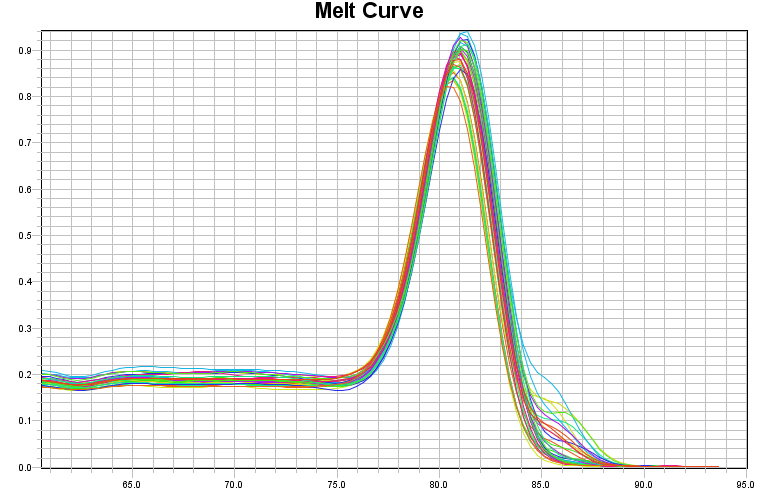
**

Real-time amplification curve and melt curve analysis for U6

**
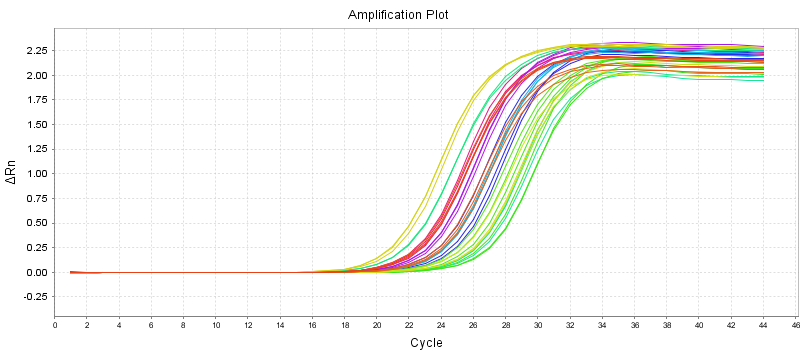

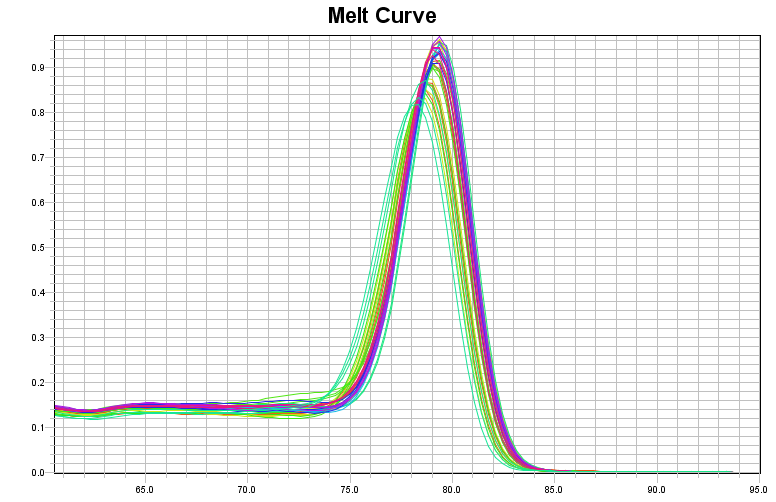
**

Real-time amplification curve and melt curve analysis for mmu_circ_0000672 between 2-month-old APP/PS1 and 8-month-old APP/PS1 mice

**
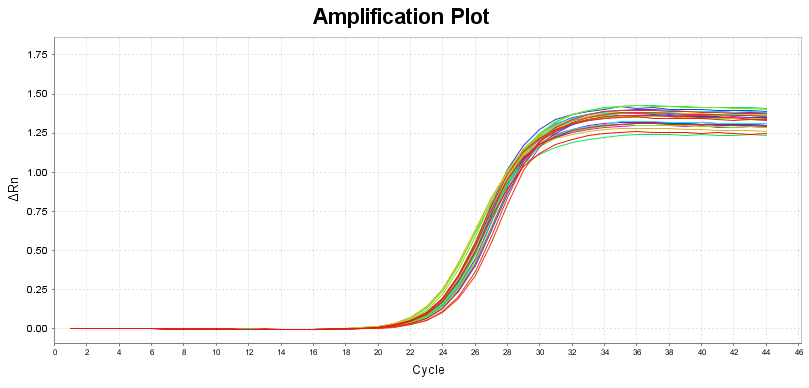

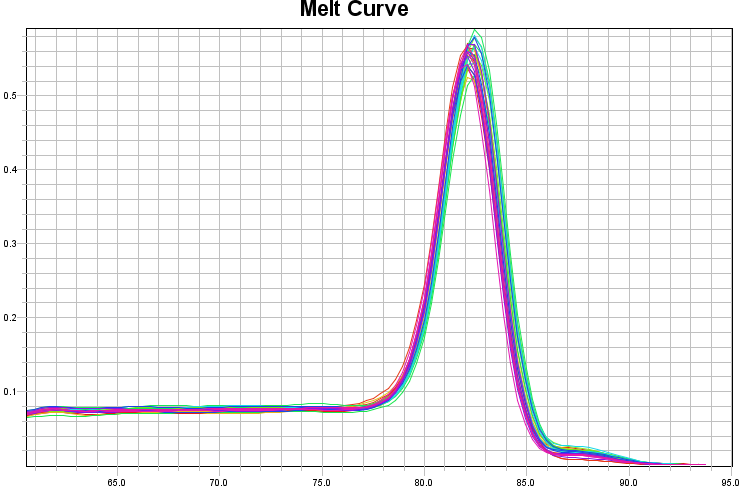
**

Real-time amplification curve and melt curve analysis for mmu-miR-344d-3-5p between 2-month-old APP/PS1 and 8-month-old APP/PS1 mice

**
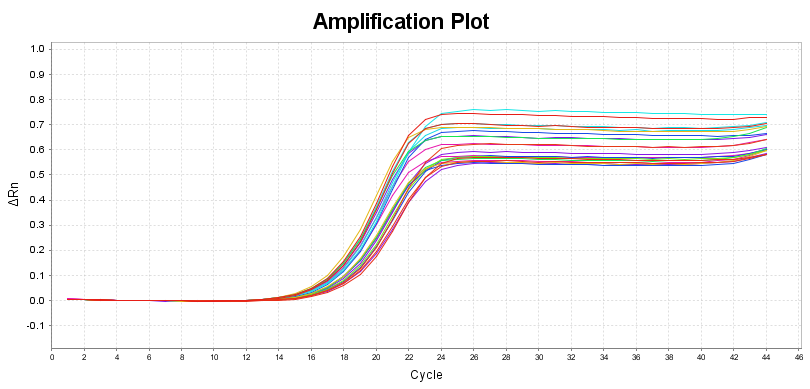

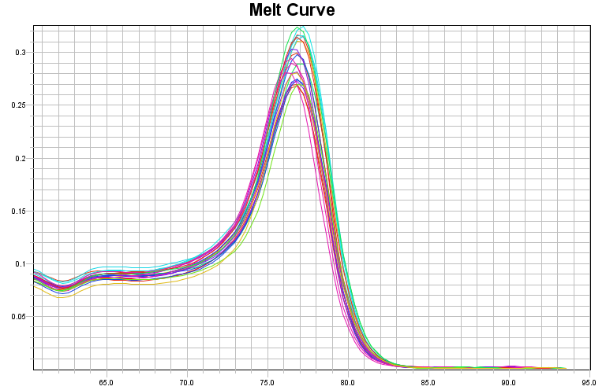
**

Real-time amplification curve and melt curve analysis for ENSMUSG00000068129 between 2-month-old APP/PS1 and 8-month-old APP/PS1 mice

**
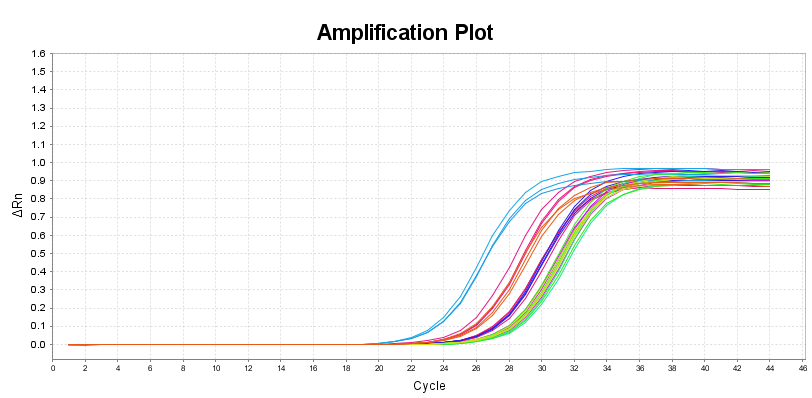

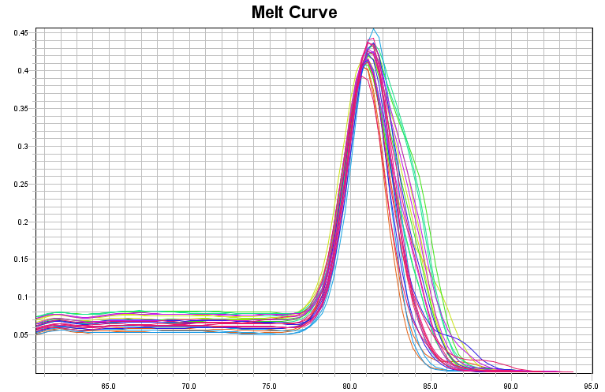
**

Real-time amplification curve and melt curve analysis for U6

**
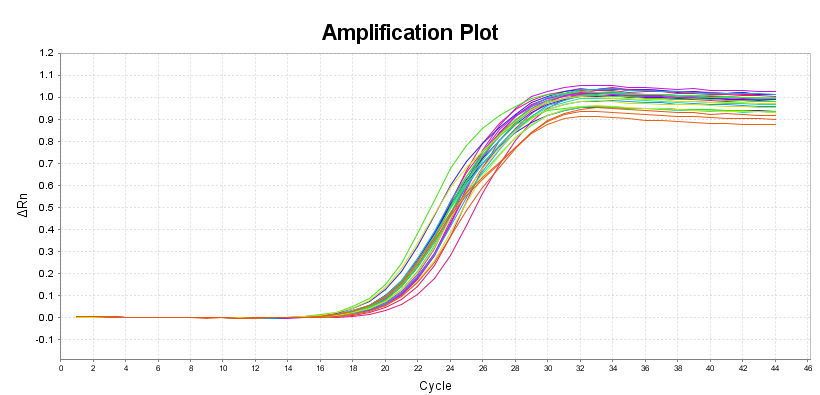

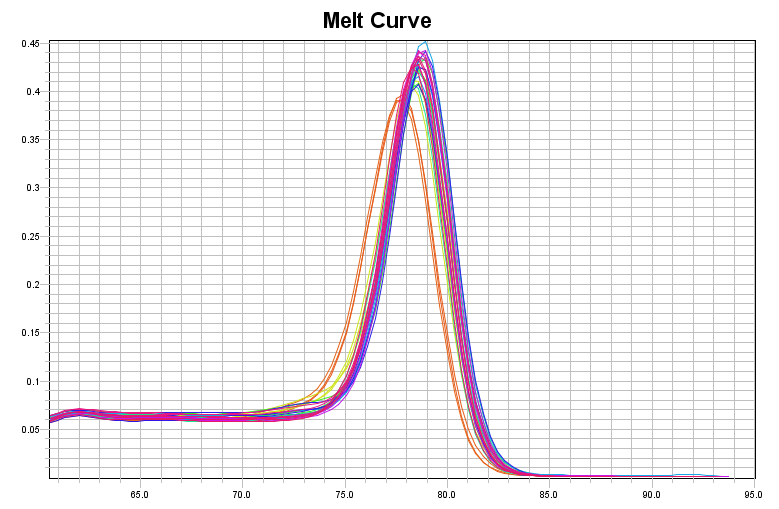
**

Real-time amplification curve and melt curve analysis for GAPDH

**
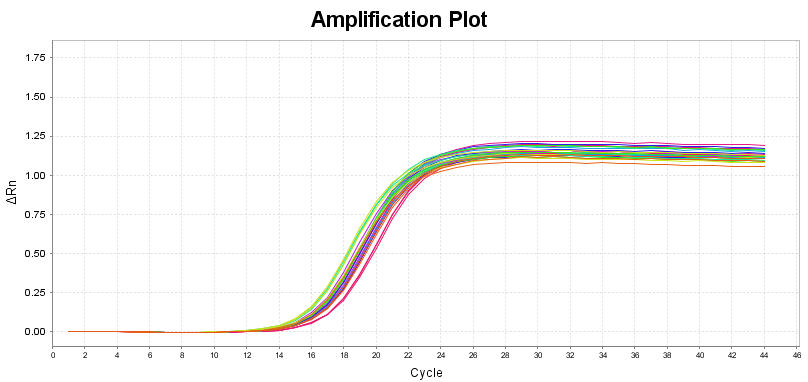

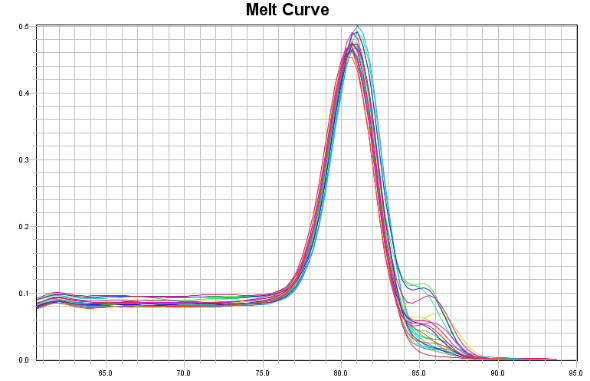
**

Real-time amplification curve and melt curve analysis for novel_circ_0003012 between 8-month-old APP/PS1 and WT mice

**
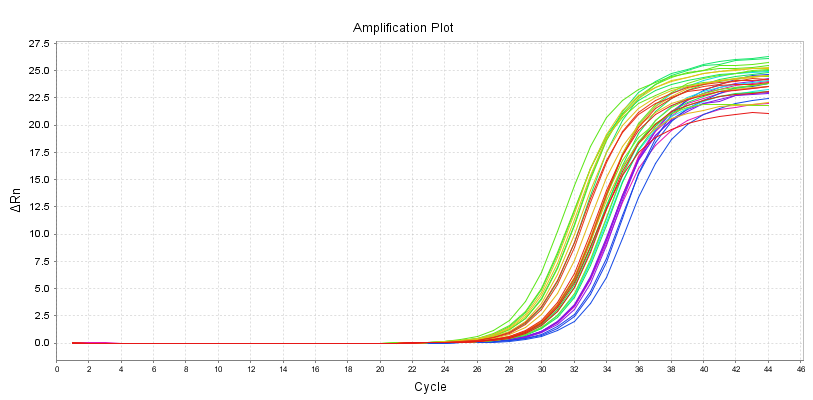

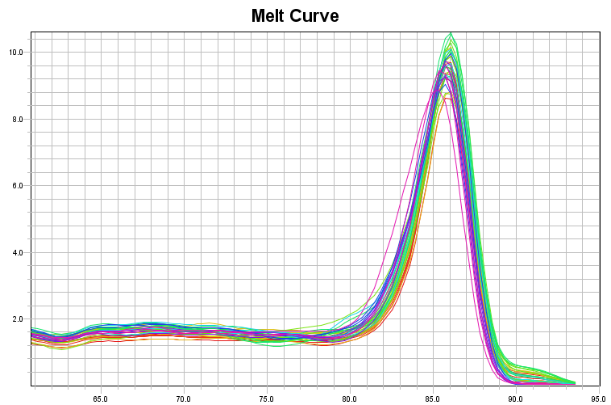
**

Real-time amplification curve and melt curve analysis for GAPDH

**
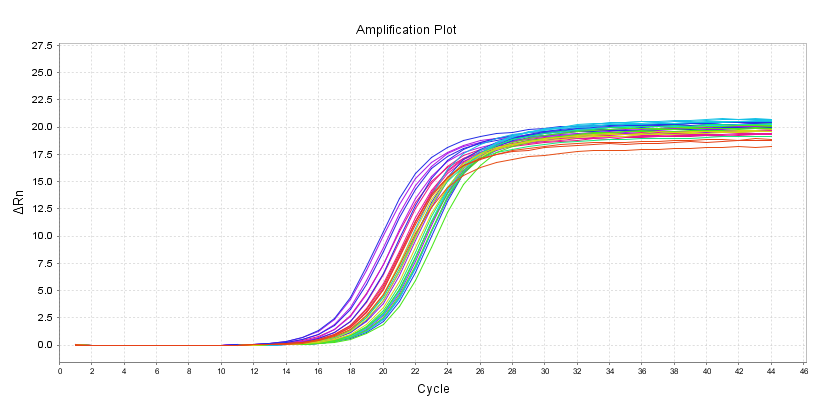

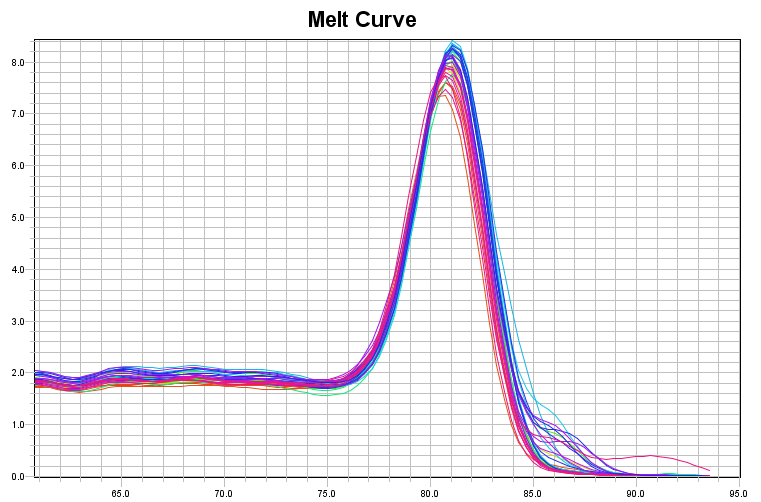
**

Real-time amplification curve and melt curve analysis for Smoc2

**
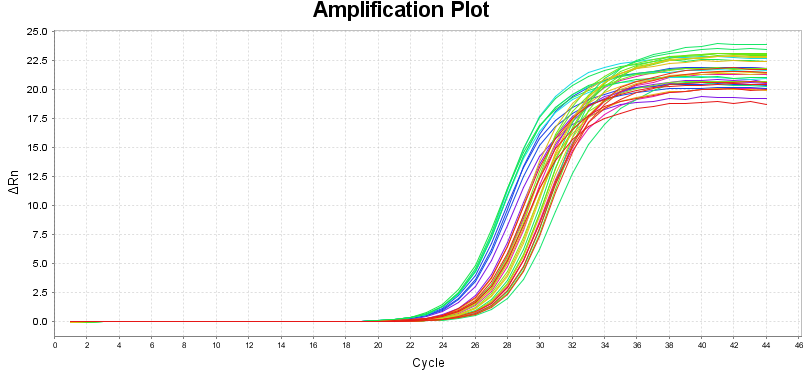

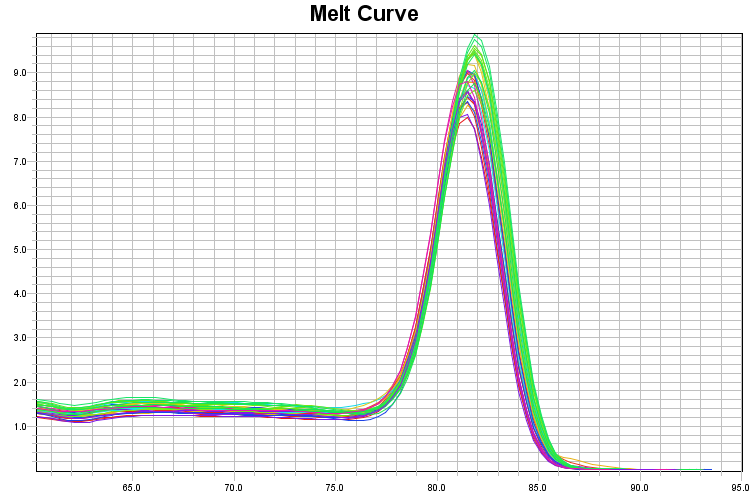
**

Real-time amplification curve and melt curve analysis for GAPDH

**
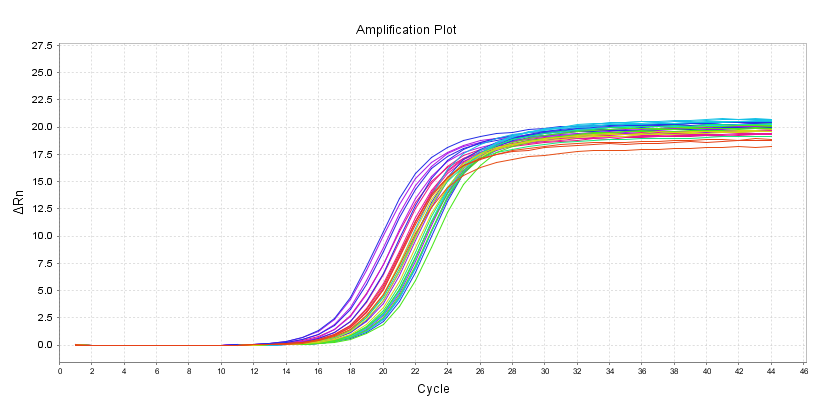

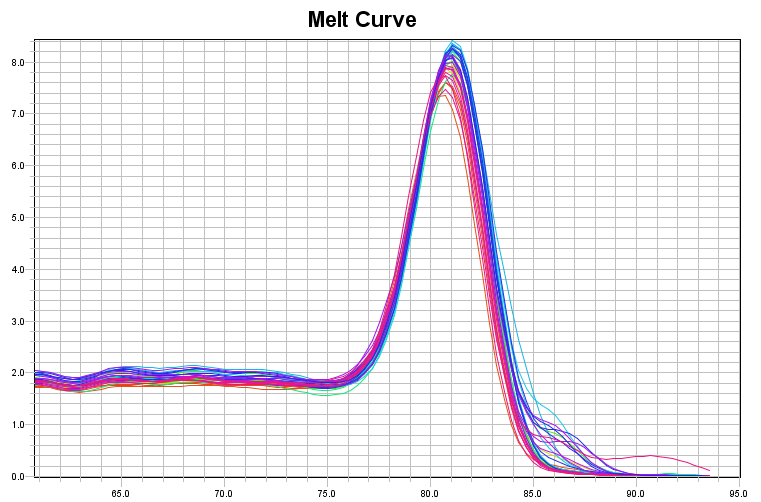
**

**Supplement Legends:**

**Supplement Figure 1.** Gene Ontology (GO) Enrichment analysis and Enriched Kyoto Encyclopedia of Genes and Genomes (KEGG) of miRNAs. (A) GO annotations of pathological progression of AD: Biological Process (BP), Cellular Component (CC), and Molecular Function (MF). Signiﬁcantly enriched GO pathways featured p values <0.05. (B) Signiﬁcantly Enriched Kyoto Encyclopedia of Genes and Genomes (KEGG) of miRNAs. Signiﬁcantly enriched KEGG pathways featured p values <0.05. Each line represents a gene, and the number of lines indicates the genes enriched.

**Supplement Figure 2.** Gene Ontology (GO) Enrichment analysis and Enriched Kyoto Encyclopedia of Genes and Genomes (KEGG) of mRNAs. (A) GO annotations of pathological progression of AD: Biological Process (BP), Cellular Component (CC), and Molecular Function (MF). Signiﬁcantly enriched GO pathways featured p values <0.05. (B) Signiﬁcantly Enriched Kyoto Encyclopedia of Genes and Genomes (KEGG) of mRNAs. Signiﬁcantly enriched KEGG pathways featured p values <0.05. Each line represents a gene, and the number of lines indicates the genes enriched.
