# Supplementary material for: CircRNA-ceRNA Network Revealing the Potential Regulatory Roles of CircRNA in Alzheimer’s Disease Involved the cGMP-PKG Signal Pathway
Source: Front Mol Neurosci. 2021 May 21;14:665788. doi: 10.3389/fnmol.2021.665788 (PMC8176118; doi:10.3389/fnmol.2021.665788)
Supplement: Supplementary file 2 [file Data_Sheet_2.DOCX]

**Table S1. Primers used in qPCR analysis**

| **Acession No.** | **Primer sequence (5‘-3’)** | **Primer direction** |
| --- | --- | --- |
| mmu_circ_0000672 | GCACAGCTCGGAAAGGCAGA | forward primer |
|  | TCCAAGGCCTGGCACATTCC | reverse primer |
| novel_circ_0001016 | GGTGACTCTGGGGTGGTCCT | forward primer |
|  | AGCTCATCTTCACTGCCGCC | reverse primer |
| novel_circ_0005255 | GCCCCTCTCAACTTGTCTCCA | forward primer |
|  | TGGAAATGCCATTAGCCAGACA | reverse primer |
| novel_circ_0003012 | CCGGTTGTAGCAGGCGTCAT | forward primer |
|  | CGTCCCCTCCATCGCCATTC | reverse primer |
| mmu-miR-344d-3-5p | AGTCAGGCTAGTGGTTATACTCC | forward primer |
| mmu-miR-296-3p | GAGGGTTGGGTGGAGGCTCTCC | forward primer |
| mmu-miR-222-3p | AGCTACATCTGGCTACTGGGTCT | forward primer |
| mmu-miR-298-3p | GGCAGAGGAGGGCTGTTCTTCCC | forward primer |
| ENSMUSG00000068129  (Cst7) | TTCAGGATGTGGGCACGCAG | forward primer |
|  | ATCTGCTCATGCAGTCGGGC | reverse primer |
| ENSMUSG00000006179  (Prss16) | TGCCTTGTGCCTCTTTCCCG | forward primer |
|  | GGGTTCCAGCAGCAGTGTGT | reverse primer |
| ENSMUSG00000040298  (Btbd16) | ACAGACTCGCACCAAGCACC | forward primer |
|  | TGGGCGACTTCAGCACCTTG | reverse primer |
| ENSMUSG00000023886  (Smoc2) | AGCAGGGAAAGCAGATGATGC | forward primer |
|  | GCTCGGTCCAGAGTGTAGGGT | reverse primer |
| U6 | GCTTCGGCAGCACATATACTAAAAT | forward primer |
|  | CGCTTCACGAATTTGCGTGTCAT | reverse primer |
| GAPDH | CGTGTTCCTACCCCCAATGT | forward primer |
|  | TGTCATCATACTTGGCAGGTTTCT | reverse primer |
