# Supplementary figures and images for: CircRNA-ceRNA Network Revealing the Potential Regulatory Roles of CircRNA in Alzheimer’s Disease Involved the cGMP-PKG Signal Pathway
Source: Front Mol Neurosci. 2021 May 21;14:665788. doi: 10.3389/fnmol.2021.665788 (PMC8176118; doi:10.3389/fnmol.2021.665788)

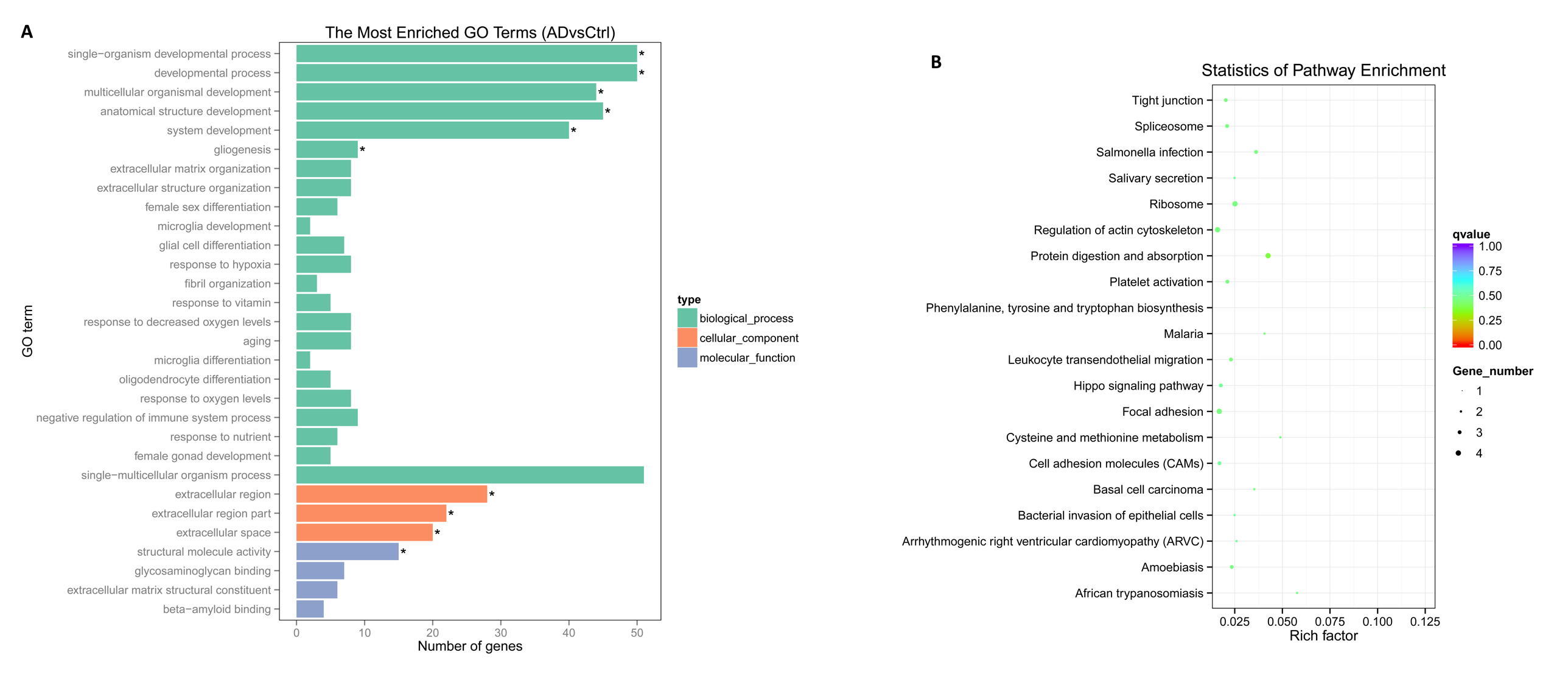

Supplement: Supplementary file 3 [file Image_1.TIF]

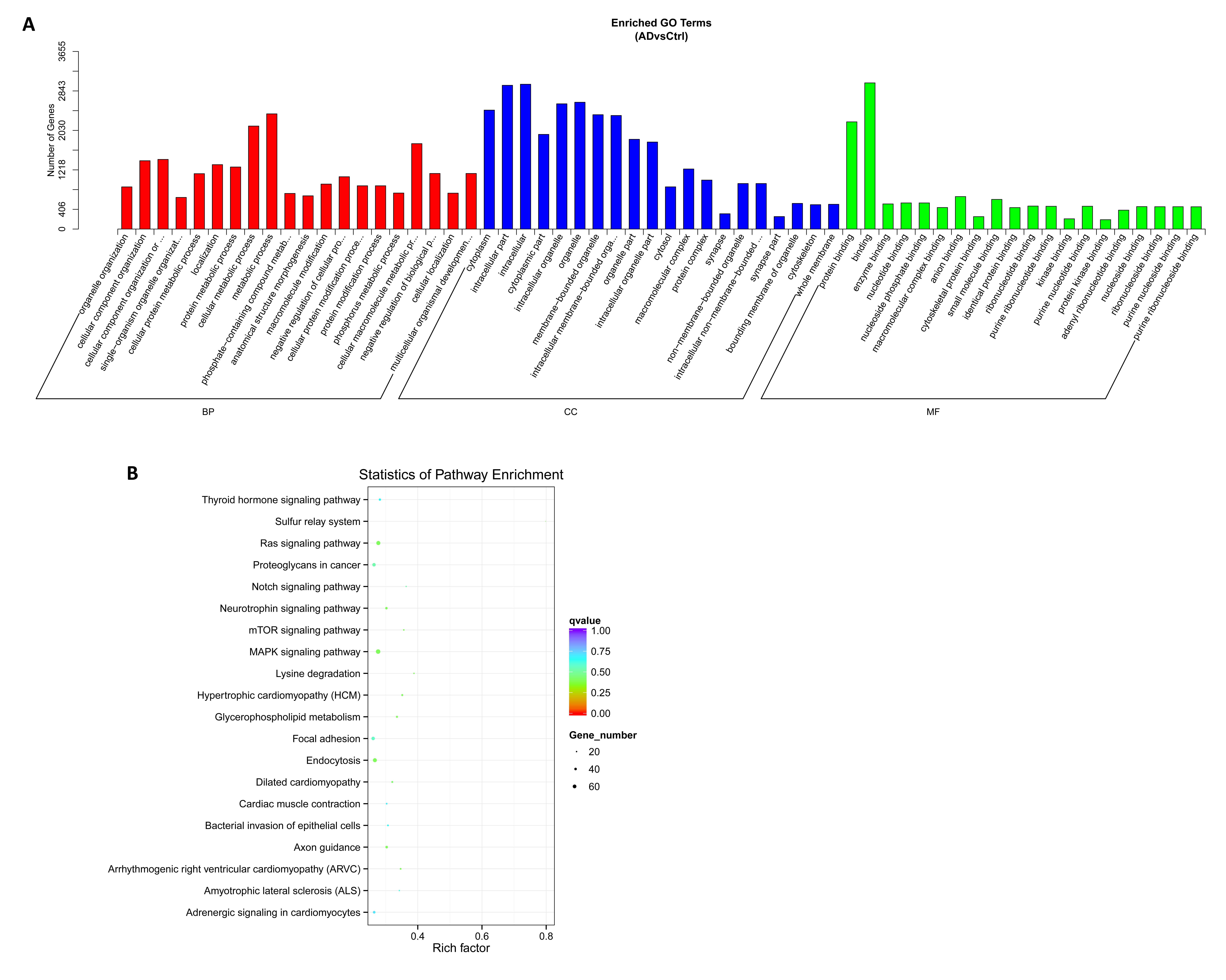

Supplement: Supplementary file 4 [file Image_2.JPEG]
